# Training Transfers the Limits on Perception from Parietal to Ventral Cortex

**DOI:** 10.1016/j.cub.2014.08.058

**Published:** 2014-10-20

**Authors:** Dorita H.F. Chang, Carmel Mevorach, Zoe Kourtzi, Andrew E. Welchman

**Affiliations:** 1 McGill Vision Research, Department of Ophthalmology, McGill University, Montreal, QC H3A 1A1, Canada; 2 School of Psychology, University of Birmingham, Edgbaston, Birmingham B15 2TT, UK; 3 Laboratory for Neuro- and Psychophysiology, KU Leuven, O&N II Herestraat 49, Box 1021, 3000 Leuven, Belgium; 4 Department of Psychology, University of Cambridge, Downing Street, Cambridge CB2 3EB, UK

## Abstract

Visually guided behavior depends on (1) extracting and (2) discriminating signals from complex retinal inputs, and these perceptual skills improve with practice [1]. For instance, training on aerial reconnaissance facilitated World War II Allied military operations [2]; analysts pored over stereoscopic photographs, becoming expert at (1) segmenting pictures into meaningful items to break camouflage from (noisy) backgrounds, and (2) discriminating fine details to distinguish V-weapons from innocuous pylons. Training is understood to optimize neural circuits that process scene features (e.g., orientation) for particular purposes (e.g., judging position) [3, 4, 5, 6]. Yet learning is most beneficial when it generalizes to other settings [7, 8] and is critical in recovery after adversity [9], challenging understanding of the circuitry involved. Here we used repetitive transcranial magnetic stimulation (rTMS) to infer the functional organization supporting learning generalization in the human brain. First, we show dissociable contributions of the posterior parietal cortex (PPC) versus lateral occipital (LO) circuits: extracting targets from noise is disrupted by PPC stimulation, in contrast to judging feature differences, which is affected by LO rTMS. Then, we demonstrate that training causes striking changes in this circuit: after feature training, identifying a target in noise is not disrupted by PPC stimulation but instead by LO stimulation. This indicates that training shifts the limits on perception from parietal to ventral brain regions and identifies a critical neural circuit for visual learning. We suggest that generalization is implemented by supplanting dynamic processing conducted in the PPC with specific feature templates stored in the ventral cortex.

## Highlights

• Before training, dorsal, but not ventral, rTMS disrupts perception in noisy displays
• Training on fine differences boosts feature templates, facilitating detection in noise
• After training, ventral, but not dorsal, rTMS becomes disruptive for targets in noise
• Functional reweighting of dorsal and ventral circuits supports learning transfer

## Teaser

Using TMS in human subjects, Chang et al. reveal differential roles of parietal and ventral cortex in extracting and discriminating visual targets, respectively. Training on fine discriminations causes a reweighting of the circuit: following learning, ventral, not parietal, activity determines target extraction task performance.

## Results

We sought to identify the cortical circuits critically involved in (1) extracting signals and (2) discriminating features, and thereafter to determine how training modifies these circuits. We targeted these perceptual processes using two tasks that rely on them differentially: (1) a signal-in-noise task that involves extracting a target masked by noise versus (2) a feature-difference task that involves judging fine differences. We were particularly interested in generalization between tasks that—according to theoretical models [1, 10]—results from the optimization of distinct processing related to (1) filtering nonrelevant items from displays and (2) reading out representations of trained features. Although considerable behavioral evidence supports this framework [11], its neural basis is uncertain, as work on the neural basis of perceptual learning has typically trained and tested on the same task and stimuli, meaning that the stratified processes supporting learning could not be separated. One exception [12] demonstrated that neural activity associated with signal-in-noise judgments became unlinked to perceptual performance following training on a feature-difference task; however, the neural circuits involved in posttraining generalization were not revealed.

**Figure 1:**
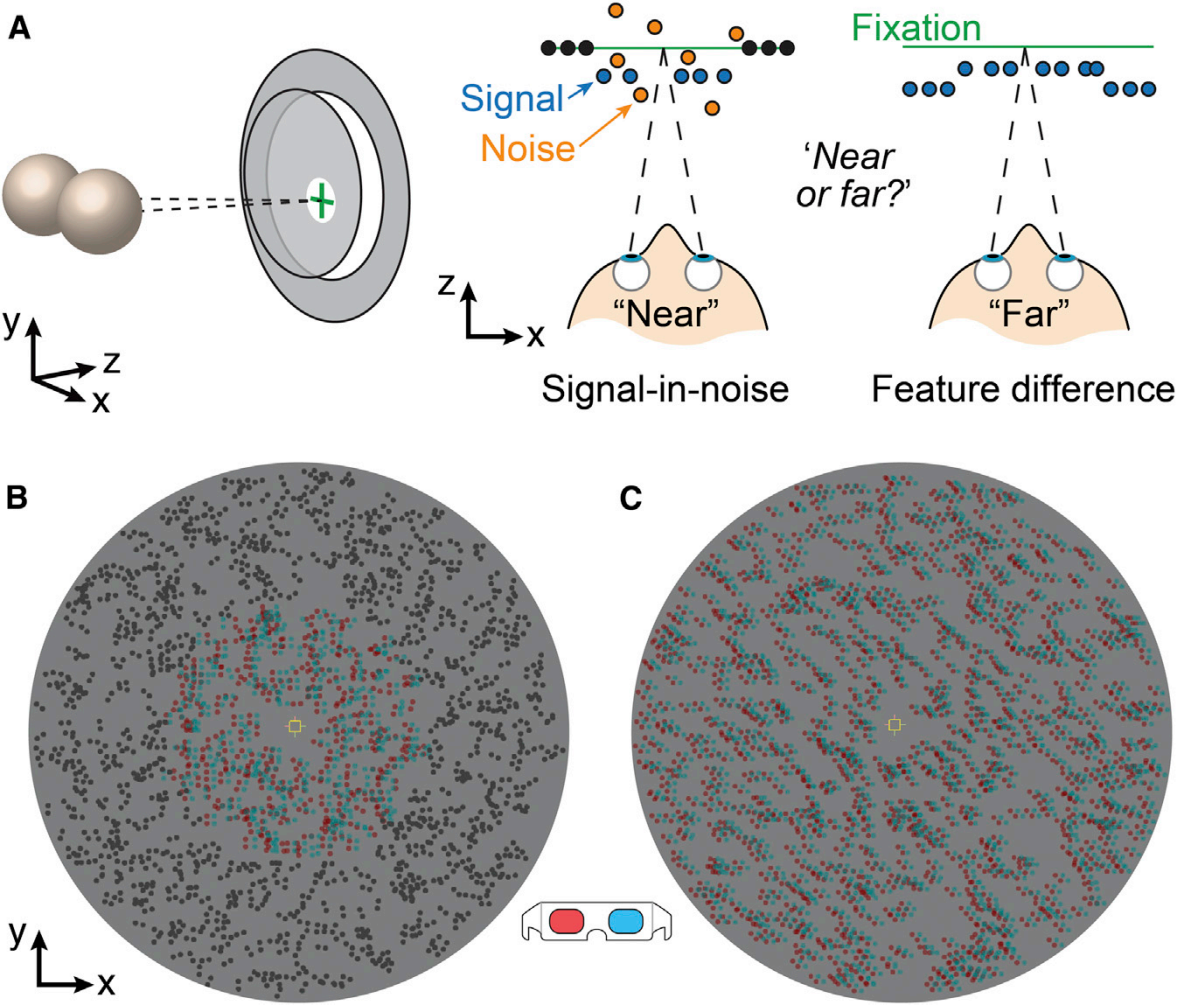
Stimuli for the Signal-in-Noise and Feature-Difference Tasks (A) Cartoon illustrations of the stimuli: the two eyes view a center-surround display. Signal-in-noise task: the target disparity was fixed at ±6 arcmin, and we varied the proportion of target signal dots relative to noise dots with randomly chosen disparities within ±12 arcmin. Feature-difference task: the disparity difference between the center and surround (±12 arcmin) was varied in fine steps. For both tasks, the participant decided whether the center is nearer or farther than the surround. (B and C) Sample random dot stimuli rendered as red-cyan anaglyphs for the signal-in-noise (B) and feature-difference tasks (C). The center was 6° in diameter, and the surround was 12° in diameter. Participants fixated on the small square marker at the center of the display.

Participants viewed a 3D display (Figure 1) and judged whether the central target was in front or behind the surrounding annulus. In the signal-in-noise task, we varied the proportion of dots defining the target plane relative to distracting dots with randomly chosen depths. In the feature-difference task, we titrated the disparity between the center and surround under noise-free presentation. We measured discrimination thresholds by adaptively controlling either the (1) signal-to-noise ratio or (2) disparity, thereby ensuring that task difficulty was equated between tasks (and before versus after training). We then used training across tasks to track changes in both perceptual performance and the neural substrates. Previous work demonstrated asymmetric transfer between tasks: training on a feature-difference task improves signal-in-noise task performance, but not vice versa [1, 7, 10, 13, 14]. We thus focused on training the feature-difference task that supports transfer.

**Figure 2:**
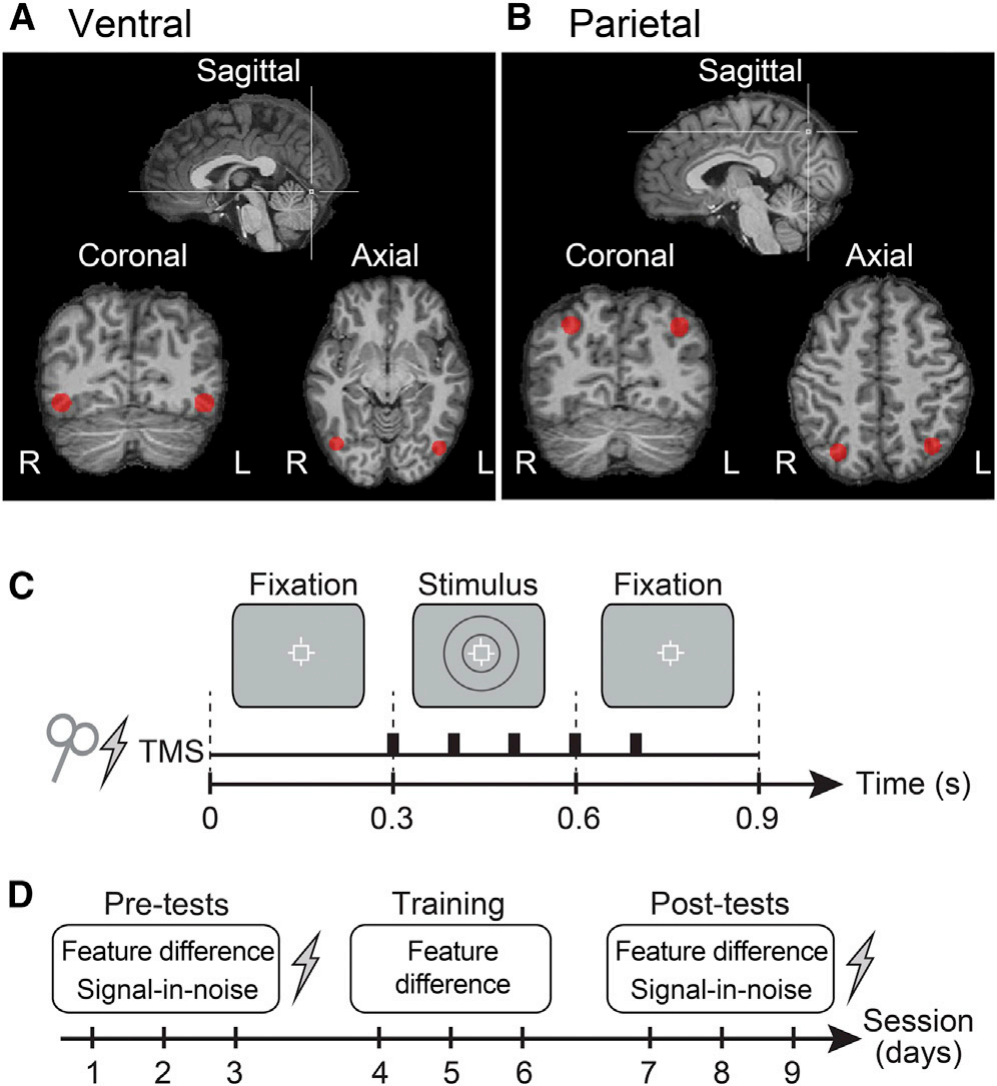
Stimulation Sites and Experimental Design (A and B) Anatomical locations of ventral (A) and parietal (B) stimulation sites. LO was defined based on functional magnetic resonance imaging (fMRI) activations; left and right PPC were identified using MRI scans with cod liver oil capsules positioned at P3 and P4 of the 10–20 electroencephalography coordinate system. (C) Stimulus presentation and TMS timeline. Online stimulation was given at 10 Hz (five pulses synchronized with stimulus onset) with a fixed intensity of 60% of the stimulator’s maximum output. (D) Experimental protocol: pretraining TMS tests (3 days), training (3 days), and posttraining TMS tests (3 days). During pre- and posttesting sessions, participants performed both tasks, but rTMS was delivered during only one of the tasks. The order of stimulation sites was counterbalanced across participants, but was fixed between pre- and posttraining tests for each observer. For each test and training run, task difficulty was adjusted by varying the stimulus according to two interleaved staircases determining thresholds at the 82%-correct level. See also Figure S2.

To probe the neural circuits involved, we used repetitive transcranial magnetic stimulation (rTMS) to temporarily disrupt processing in candidate regions of interest. We were a priori interested in posterior parietal cortex (PPC) that is involved in top-down attentional selection of targets in noise [15] by means of figure-ground segmentation [16] and learning [17], in contrast to ventral areas that process disparity-defined forms [18, 19] and feature templates [20]. We therefore measured performance on (1) feature-difference and (2) signal-in-noise tasks while participants received rTMS over PPC (dorsal) or lateral occipital (LO) (ventral) areas (Figure 2; Table S1 available online). To control for generalized interference from rTMS, we stimulated a control site (Cz) to provide a baseline for psychophysical performance (Figure S1A shows raw thresholds).

**Figure 3:**
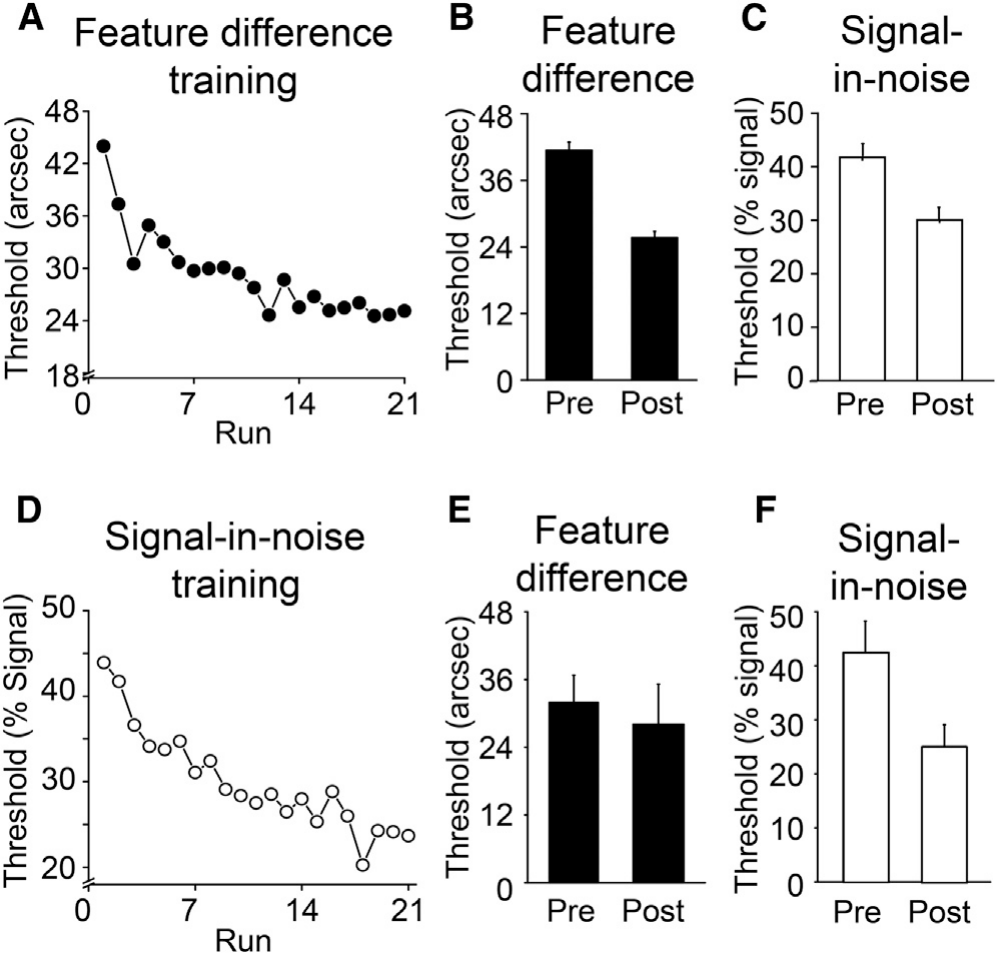
Behavioral Thresholds before and after Training (A) Threshold changes across the 21 training runs (2,184 trials) of the feature-difference task. (B and C) Mean performance for the feature-difference and signal-in-noise discrimination tasks, before and after training, pooled across participants (n = 27). (D) Threshold changes across the 21 training runs (2,184 trials) of the signal-in-noise task. (E and F) Mean performance for the feature-difference and signal-in-noise discrimination tasks, before and after training, pooled across participants (n = 6). Error bars represent ±1 SEM. (Note that mean pretraining thresholds for participants in B are slightly higher than for the participants in E; however, selecting participants from the feature-difference training group, B, with comparable pretraining thresholds to the signal-in-noise training group, E, revealed a clear training effect, suggesting that the lack of significant transfer for participants trained on the signal-in-noise task could not be ascribed to a floor effect whereby it was not possible for thresholds to improve further.)

Before considering the rTMS results, we confirmed the asymmetric transfer between tasks [7, 13] by considering participants’ performance on both the trained and untrained tasks before and after 3 days of training on one of the tasks. Training on the feature-difference task (Figure 3A) improved performance on both the feature-difference task (Figure 3B; *t*_26_ = 8.79, p < 0.001) and the signal-in-noise task (Figure 3C; *t*_26_ = 9.18, p < 0.001). By contrast, training on the signal-in-noise task (Figure 3D) benefited this task (Figure 3E; *t*_5_ = 3.67, p = 0.014) but there was no transfer to the feature-difference task (Figure 3F; *t*_5_ < 1, p = 0.55). This asymmetry was supported by an rANOVA with a significant interaction of session (pre versus post) and training task (feature-difference versus signal-in-noise) for the feature task (*F*_1,31_ = 6.60, p = 0.015), but not the signal-in-noise task (*F*_1,31_ = 2.52, p = 0.122).

**Figure 4:**
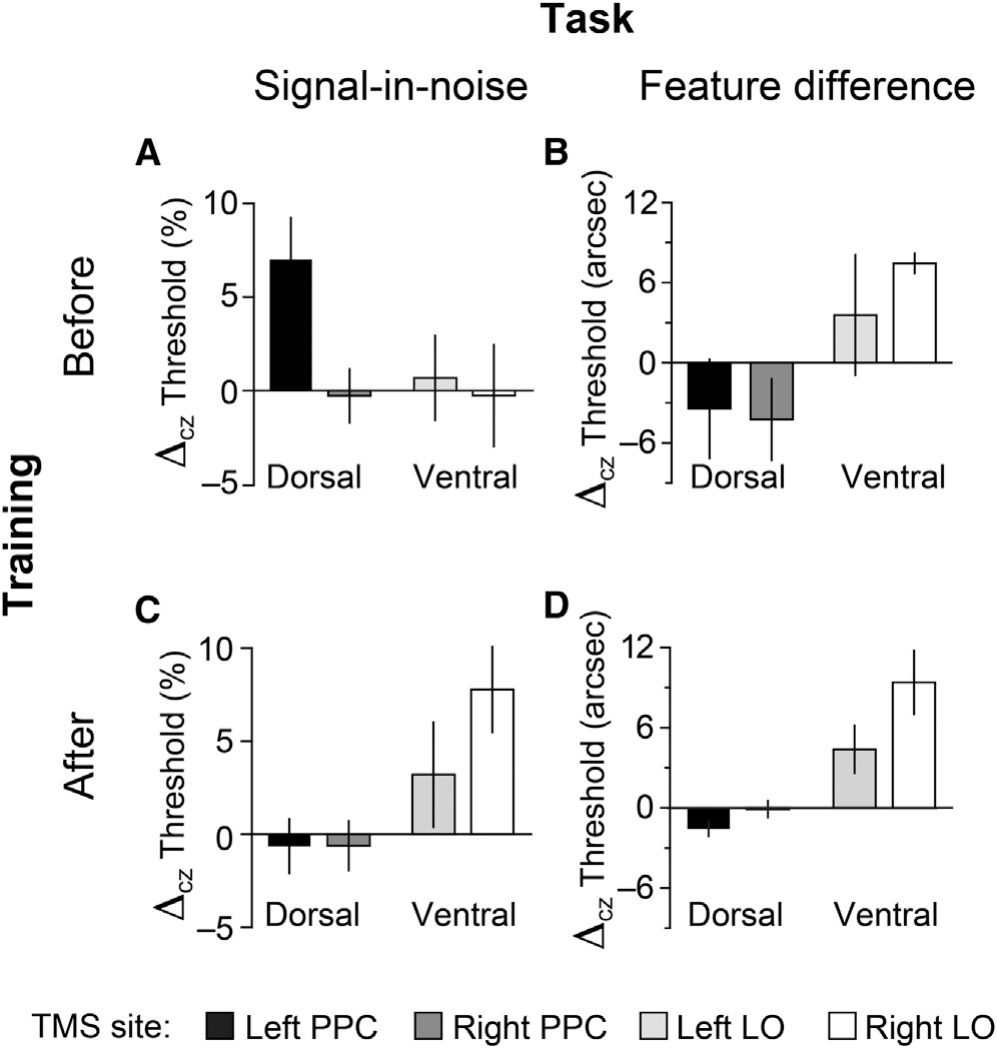
The Effects of Dorsal and Ventral rTMS on Signal-in-Noise and Feature-Difference Task Performance before versus after Training (A) Mean performance (relative to Cz baseline) for the signal-in-noise task before training with rTMS over dorsal (n = 12) or ventral (n = 12) areas; see Figure S1 for unnormalized thresholds. (B) Mean performance (relative to Cz baseline) for the feature-difference task before training with rTMS over dorsal (n = 6) or ventral (n = 6) areas. (C) Mean performance for the signal-in-noise task after training with rTMS over dorsal or ventral areas. (D) Mean performance for the feature-difference task after training with rTMS over dorsal or ventral areas. Error bars represent ±1 SEM. See also Figures S1, S3, and S4.

Considering the results of rTMS before training, we found worse performance (i.e., higher thresholds) for left PPC rTMS than Cz, in contrast to LO stimulation, where performance was unaffected (Figure 4A). rANOVAs conducted on the raw discrimination thresholds indicated a significant difference between stimulation sites (Cz, left PPC, right PPC) for dorsal rTMS (*F*_1,11_ = 9.79, p = 0.01), in contrast to no significant differences (*F*_2,22_ < 1, p = 0.915) between sites (Cz, left LO, right LO) for ventral stimulation. The effect in dorsal cortex was specific to the left PPC (*t*_11_ = 3.13, p = 0.009) and replicable (Figure S1C). This left lateralization was anticipated, because noise dots were distracting: damage to left parietal cortex impairs patients’ abilities to ignore salient distracting information [21], whereas healthy adults are poorer at inhibiting high-salience distracters during TMS over left PPC [22].

We found contrasting results for the pretraining tests on the feature-difference task (Figure 4B). In particular, PPC stimulation did not affect judgments (*F*_2,10_ < 1, p = 0.446) but rTMS to LO did (*F*_2,10_ = 9.18, p = 0.005). This LO effect was more pronounced in the right hemisphere (although not statistically significant). These dissociated results between dorsal and ventral areas for signal-in-noise and feature tasks suggest distinct contributions to perception: left parietal cortex may be critically involved in external noise filtering, whereas feature representations in ventral LO may support fine discriminations.

Following the pretraining sessions, we tested whether training on the feature-difference task caused changes in the neuronal circuits supporting perceptual judgments. Given that feature-difference training promotes transfer to the signal-in-noise task, it was of critical interest to determine the role of ventral circuits in the signal-in-noise task after training. In contrast to the pretraining results, we found that signal-in-noise task performance was unaffected by left (or right) PPC stimulation (*F*_2,22_ < 1, p = 0.869). Strikingly, performance was instead significantly (*F*_2,22_ = 5.27, p = 0.01) worse under ventral stimulation (Figure 4C).

This reversal of rTMS-induced deficits for the signal-in-noise task from dorsal to ventral cortex was supported by a significant three-way interaction (rANOVA, *F*_2,44_ = 6.40, p = 0.004) between training session (pre versus post), location (dorsal versus ventral), and stimulation site (left hemisphere, right hemisphere, Cz). Importantly, a significant interaction (*F*_1.6,35.2_ = 9.06, p = 0.001) between session and location confirmed the dissociable role of these areas in the signal-in-noise task before versus after training. A follow-up two-way rANOVA showed a significant interaction of training and site (left PPC, right PPC, Cz) (*F*_2,22_ = 9.76, p = 0.001), consistent with decreased performance before, but not after, training for left PPC. For ventral rTMS, a significant interaction of training and site (left LO, right LO, Cz) (*F*_2,22_ = 6.29, p = 0.007) was also observed, but the pattern was reversed: performance under ventral stimulation decreased after, but not before, training. The effect in ventral cortex for the signal-in-noise task after training was stronger in the right hemisphere (*t*_11_ = 2.23, p = 0.048), echoing the pretraining results for performance on the feature-difference task.

These dissociable effects of rTMS suggest a fundamental change in the cortical areas that limit performance on the signal-in-noise task, such that there is a decreased contribution of parietal cortex and an increased role of LO after training. Further testing revealed that ventral sites remained important for the feature-difference task: LO stimulation after training remained disruptive for feature-difference judgments (Figure 4D; main effect of stimulation site: *F*_2,10_ = 9.18, p = 0.005, but no interaction with training: *F*_2,10_ < 1, p = 0.901). In common with the preceding results, rTMS effects were stronger in right LO (*t*_5_ = 2.61, p = 0.047).

We next asked whether changes in the circuit involved in signal-in-noise identification depend on training on the feature-difference task. We first tested participants (n = 8) on the signal-in-noise task before and after 3 days of rest (Figure S2A). We found that parietal stimulation remained disruptive when participants were not actively trained: there was a main effect (*F*_1,7_ = 27.09, p = 0.001) of site (left PPC, Cz) but no interaction with session (*F*_1,7_ < 1, p = 0.664). Second, we trained new participants (n = 6) on the signal-in-noise task rather than the feature-difference task (Figure S2B). We found that ventral stimulation had no effect on signal-in-noise task performance (before or) after training on this task (*F*_1,5_ < 1, p = 0.84), indicating that feature-difference training was critical. This could not be due to insufficient training for the signal-in-noise task, because learning rates were matched (Figure S2C) [13]. We speculate that after signal-in-noise training, information in earlier visual areas may be critical, because the high spatial resolution of earlier sensory neurons affords refined signal-in-noise discrimination following coarser target detection at higher processing stages [23]. Third, we retested available participants trained on the feature-difference task 1–6 months after initial testing. We found that shifts in the cortical loci limiting signal-in-noise judgments lasted for a long period: ventral (rather than dorsal) stimulation retained its disruptive effect for each individual participant (Figure S1D). Taken together, these results suggest that training on feature differences changes the functional contributions of dorsal and ventral cortex for perceptual judgments in noisy displays. This functional reweighting of the circuit involved in target identification from noise is specific and longer term in nature, requiring training on a task designed to boost feature templates.

To make a direct comparison between tasks measured in different units, we computed percent change in threshold before versus after training (although note that this approach is not without complication [13, 24]). We found a significant interaction between location, task, and rTMS site (*F*_1,29_ = 4.39, p = 0.045), highlighting dissociable effects between tasks before versus after training. This dissociated pattern of results made experimental artifacts unlikely. First, nonspecific improvements in task performance could not explain our findings, because generalization was asymmetric. Second, the adaptive psychophysical procedure ensured that difficulty was equated for the different tasks before and after training, ruling out explanations based on general attentional demands. Third, any differences in rTMS efficacy between sites could not account for differences before versus after training. Further, performance disruption was comparable for PPC TMS before training and LO TMS after training. Fourth, we tested whether rTMS to dorsal versus ventral areas during pretraining sessions might interfere differentially with participants’ ability to learn on subsequent days. We found no differences in the total learned (*F*_2,28_ < 1, p = 0.48) or learning rate (*F*_2,28_ < 1, p = 0.43) for participants who received no stimulation or rTMS of different sites (Figure S2D). Fifth, measuring binocular eye movements during rTMS showed that stimulation did not disrupt eye movement control (critical for stereopsis): eye vergence was stable and not systematically affected by rTMS (Figure S3), consistent with a previous report [25]. Finally, we analyzed participants’ response times (Figure S4); these quickened following training (signal-in-noise task, *F*_1,22_ = 6.02, p = 0.023; feature-difference task, *F*_1,7_ = 7.29, p = 0.031) but did not differ between sites, as expected for threshold measurement tasks where participants were instructed to be as accurate as possible.

## Discussion

Here we provide evidence for functional reweighting of a circuit that supports perceptual judgments through training. We tested performance on two tasks that differentiate the stages optimized during perceptual learning [1, 10]. This allowed us to identify cortical loci that limit performance on (1) signal extraction and noise filtering versus (2) the representation of features. These fundamental perceptual abilities critically depend on the dynamic processing capacities of the PPC versus template storing in ventral cortex. Thereafter, we showed that training designed to boost feature templates changes the loci that limit task performance: the signal-in-noise task is no longer critically limited by parietal activity but rather by ventral cortex. This identifies a cortical basis for theoretical models of learning that posit that feature-difference training optimizes the readout of feature templates [1, 10, 26].

Previous electrophysiological [27, 28, 29] and neuroimaging studies [30, 31, 32, 33] focused on changes in neural responses for a given task and stimulus set following dedicated training on that task and stimulus set. However, this does not allow the computational stages involved to be differentiated, because optimization could take place at multiple levels. Here we took the approach of contrasting two tasks that rely differentially (but not exclusively) on (1) signal extraction and (2) feature discrimination. By examining how training affects performance not only on the trained task but also on a different untrained one, we uncover the cortical basis of the hypothesized mechanisms [7, 13]. We propose that performance in both the signal-in-noise task and the feature-difference task engages parietal and ventral loci but that the extent of activation differs. An observer’s judgment can be no better than the noisiest estimation stage. For the signal-in-noise task, parietal cortex initially imposes this limit on performance. However, following training, readout weights are optimized [10]; in consequence, feature representations in ventral cortex become the limiting stage that determines task performance.

Previously, Chowdhury and DeAngelis [12] showed that generalized training from a feature task reduced the involvement of MT/V5 on a signal-in-noise task: reversible inactivation disrupted the monkey’s perceptual judgments before, but not after, training. Similarly, TMS over human PPC can produce perceptual interference before, but not after, training [34], suggesting that the effects of dorsal stimulation can diminish following training. Our results support this idea; however, this work did not identify the loci responsible for posttraining performance. Critically, we demonstrate that ventral circuits support signal-in-noise task performance after training, indicating that learning changes the limits on visual perception from the posterior parietal to the ventral cortex. We assessed the involvement of hMT+/V5 under our paradigm, testing new observers (n = 6) on the signal-in-noise task before and after training on the feature-difference task. We found no interference on task performance from rTMS before or after training (Figure S1E). This difference from the macaque likely reflects the absence of motion from our stimuli.

We conjecture that training on fine differences optimizes the representations of disparity features in LO, consistent with evidence for disparity processing in macaque inferotemporal cortex [18, 19] and downstream V4 [35]. These boosted features facilitate figure-ground segmentation and the identification of targets in noise, diminishing the need for filtering by the parietal cortex. This process may involve augmented Hebbian reweighting, where a single set of readout weights is modified through training on feature differences [36]. Under this view, our data point to LO as the locus for these readout weights. The fact that rTMS was slightly stronger in rLO is compatible with evidence that right temporal cortex has a better capacity for template representations [37] and that right ventral TMS affects judgments of object properties [38, 39]. The maintained role of this area for feature discriminations before and after training suggests that it plays a key role in depth-perception tasks. Finally, it is likely that the cortical network involved in perceptual learning extends beyond the areas we targeted, depending on the tasks and stimuli used. Nevertheless, our findings indicate a key type of circuit reweighting for generalization through stored representations that may be applicable to other stimuli and tasks.

The brain retains considerable capacity for plasticity in adulthood. Our finding of a functional dissociation between the dorsal and ventral regions before and after training highlights changes in the functional roles of regions underlying perception. The changes we observe may represent the operation of a general processing strategy through which the brain stores information from previous experience in ventral circuits to reduce the need for dynamic processing by the dorsal stream. Thus, task generalization may paradoxically depend on bolstering specific feature representations stored in the ventral cortex. As such, there may be value in boosting feature representations to ameliorate healthy (e.g., aging [40]) and clinical (e.g., attention-deficit/hyperactivity disorder [41]; neuropsychological patients [21]) populations who show impaired ability to ignore distracting information during everyday tasks.

## Supplemental Information

Document S1. Supplemental Experimental Procedures, Figures S1–S4, and Table S1

Document S2. Article plus Supplemental Information
