# Supplementary material for: Training Transfers the Limits on Perception from Parietal to Ventral Cortex
Source: Curr Biol. 2014 Oct 20;24(20):2445–50. doi: 10.1016/j.cub.2014.08.058 (PMC4204932; doi:10.1016/j.cub.2014.08.058)
Supplement: Document S1. Supplemental Experimental Procedures, Figures S1–S4, and Table S1 [file mmc1.pdf]

**Current Biology, Volume *24***

**Supplemental Information**

# Training Transfers the Limits on Perception from Parietal to Ventral Cortex

**Dorita H.F. Chang, Carmel Mevorach, Zoe Kourtzi, and Andrew E. Welchman**

## Fig S1: Effects of parietal rTMS, retention of rLO effect, and hMT+/V5 stimulation (Fig. 4)

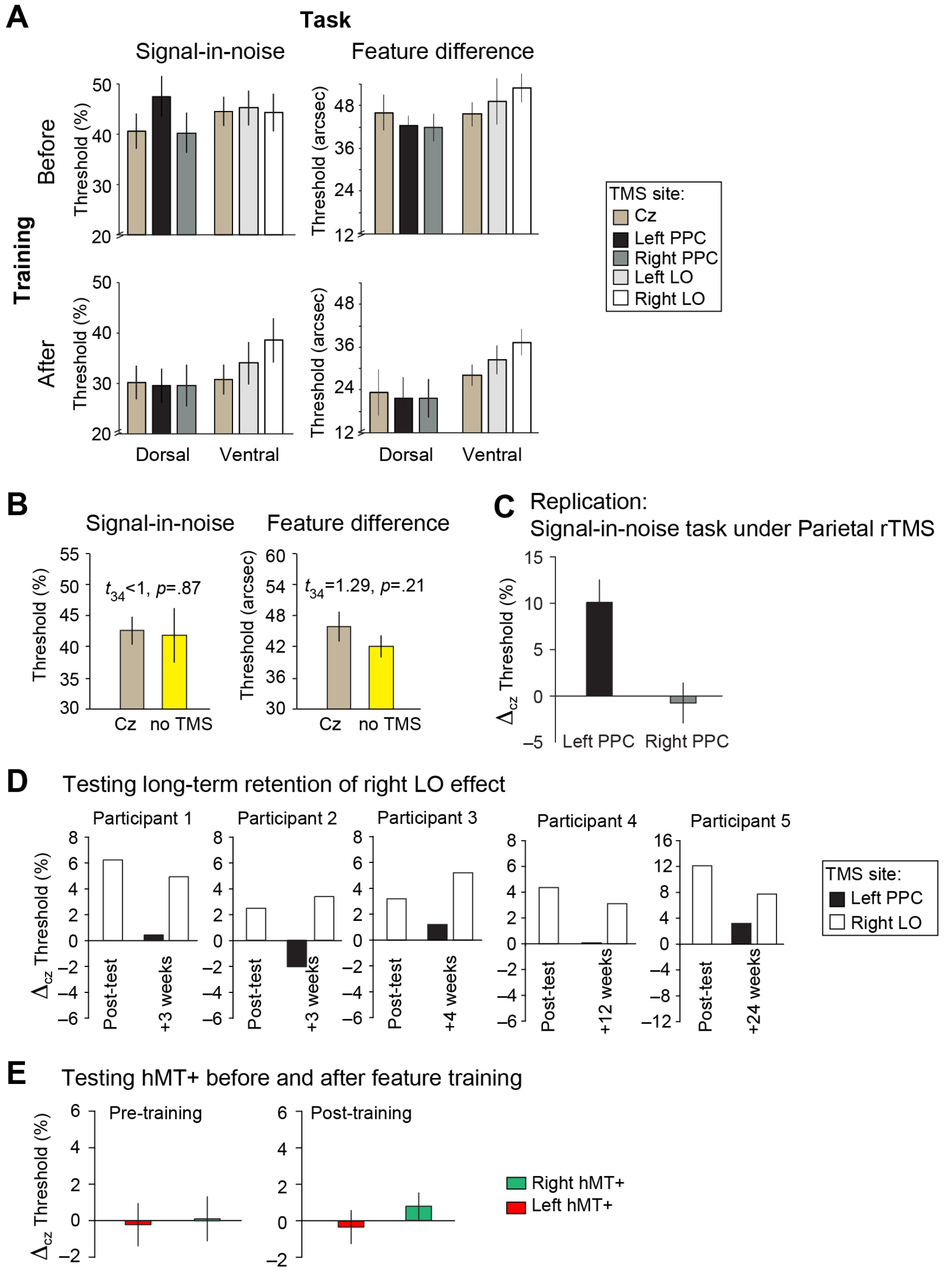


(**A**) Between-subjects average thresholds for the signal-in-noise and feature tasks during rTMS of the control (Cz) or regions of interest (PPC or LO) sites, before- and after- training. These data are presented relative to the Cz thresholds in Main Figure 4. The error bars depict the s.e.m.

(**B**) Comparison between control site (Cz) *vs*. no rTMS. Between-subjects mean thresholds are shown (with error bars for the s.e.m.). Thresholds were similar under control site TMS and when no TMS was applied.

(**C**) Replication of the parietal rTMS effect. We repeated our assessment of the effects of left PPC and right PPC stimulation (relative to Cz) during performance on the signal-in-noise ($n$=9). As in the main experiment, we observed a significant difference between stimulation over the different sites ($F_{2,16}$=12.7, $p$<.001), with pronounced threshold increases under left PPC stimulation.

(**D**) We investigated the long-term effects of training by recalling five observers at 3-4 weeks (Participants 1-3), 12 weeks (participant 4), or 24 weeks (Participant 5) following training. We retested performance on the signal-in-noise task during stimulation over left PPC, right LO and Cz (control). No additional task training was provided before these tests. For all five observers, stimulation of right LO still impaired performance, indicating long-term changes in the importance of ventral circuits.

(**E**) We tested new observers ($n$=6) on the signal-in-noise task with rTMS over right and left hMT+, and Cz, before and after training on the feature difference task. Thresholds improved after training, but were not differentially affected by hMT+/V5 stimulations versus Cz [rANOVA on site (right and left hMT+, Cz) and test (pre-, post-training); main effect of training, $F_{1,5}$=57.3, $p$<.001, but no other significant effects]. Previous work using 60% intensity stimulation affected perceptual judgments [S1], suggesting that it is unlikely that the null effect for hMT+/V5 stimulation was due to insufficient rTMS intensity.

**Fig. S2: Considerations of the role of different types of training (Main Fig.3)**

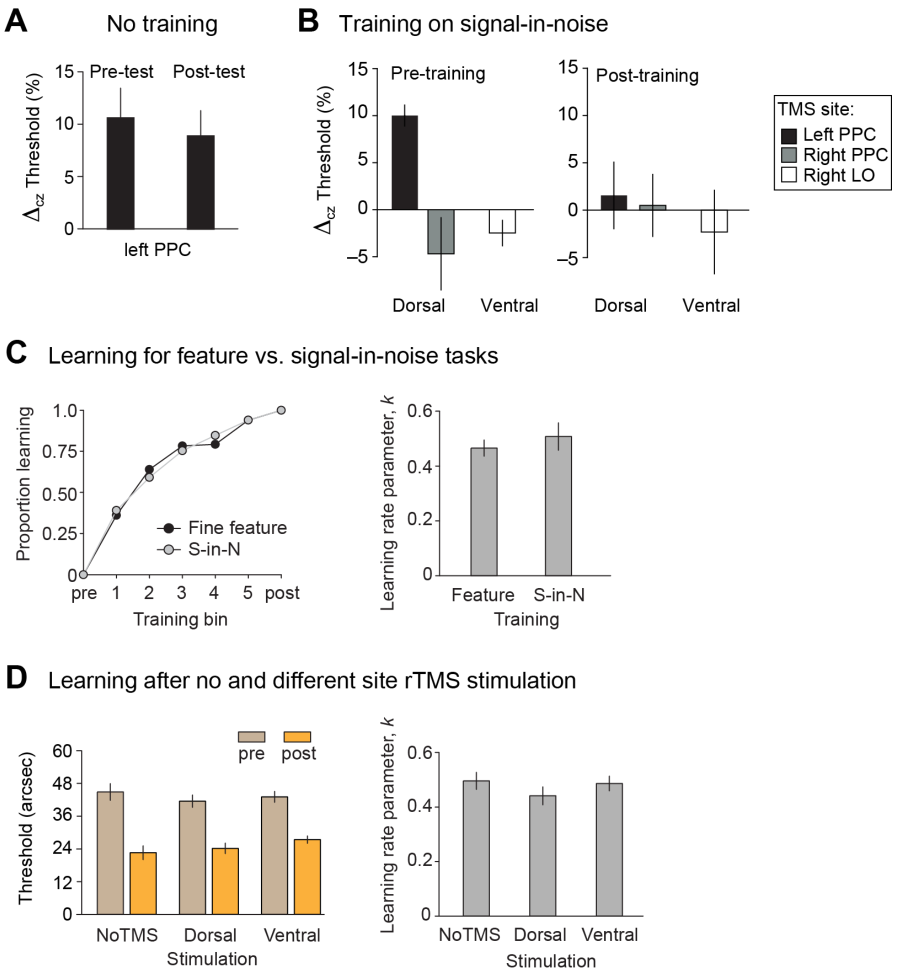


(**A**) We tested new observers ($n$=8) on the signal-in-noise task with rTMS over left PPC and Cz before and after three days of rest. Stimulations over left PPC affected thresholds significantly relative to baseline Cz both during initial tests ($t_7$=3.69, $p$=.008) and re-tests ($t_7$=3.62, $p$=.008). Additionally, thresholds relative to Cz did not differ between tests ($t_7$<1, $p$=.66) indicating that active training was required to see the reduction in the contribution of left parietal cortex to performance on the signal-in-noise task.

(**B**) We tested a new group of observers ($n$=6) on the signal-in-noise task with stimulation over Cz, left PPC, right PPC and right LO before and after three consecutive days of training on the signal-in-noise task (rather than the feature difference task which was used in training for the main experiments). We found a significant training by stimulation site interaction ($F_{3,15}$=4.62, $p$=.018) which was due to significantly worse performance for left PPC stimulation before training ($F_{3,15}$=6.5, $p$=.005) but not afterwards ($F_{3,15}$<1, $p$=.63). These data demonstrate that the perceptual contribution of the posterior parietal cortex is affected by training [S2]. Critically however, increasing the role of ventral area LO is not a general feature caused by training: without training on fine feature differences, we do not see a role for ventral areas. This suggests that training paradigms that boost feature representations may be necessary for re-weighting and transfer.

(**C**) We compared behavioural improvements for the tasks by considering the normalised learning functions for training on feature differences and the signal-in-noise task. As the tasks measure thresholds in different units—disparity vs. signal to noise proportion—we calculated normalised performance in each session as a proportion of the total amount learnt over the course of training – thus the y-axis is normalised between zero (pre-training) and one (post-training) performance. The graph depicts the mean learning function across individuals (Feature task $n$=24; signal-in-noise task $n$=6). Consistent with behavioural data presented elsewhere [S3] we found very similar learning functions for the feature difference and signal-in-noise tasks. Formally, we fit each individual's learning function with an exponential saturating learning model with the form $b = k \ln(a)$, where $a$ is the training block, $b$ is the proportion of learning and $k$ is the fitted learning rate parameter. We found no reliable difference in the learning rate parameter for participants trained on the feature task vs. the signal-in-noise task ($t_{28}$<1, $p$=.40) indicating similar levels of improvement for the two tasks.

(**D**) We tested whether rTMS on previous testing days might interfere with learning during the subsequent 3 days of training on the feature task. First we considered thresholds for the feature task before and after training for 3 groups of participants: (i) those given rTMS to PPC ($n$=12), (ii) those given rTMS to LO ($n$=12) and (iii) participants ($n$=7) who did not receive TMS (including data from four participants of Exp. 2 of Chang *et al* [S3]). There were clear improvements in task performance after training ($F_{1,28}$=103.2, $p$<.001), but no differences between the different groups ($F_{2,28}$<1, $p$=.48) nor an interaction ($F_{1,2}$=1.1, $p$=.37). In addition, we fit the learning functions for each individual and compared the rate parameter ($k$) between groups, finding no evidence for differences between groups ($F_{2,28}$<1, $p$=.43). (NB all the data presented here represent behavioural performance where at least 24 hours had elapsed since rTMS.)

## Fig. S3: Horizontal eye vergence response functions (Main Fig. 4)

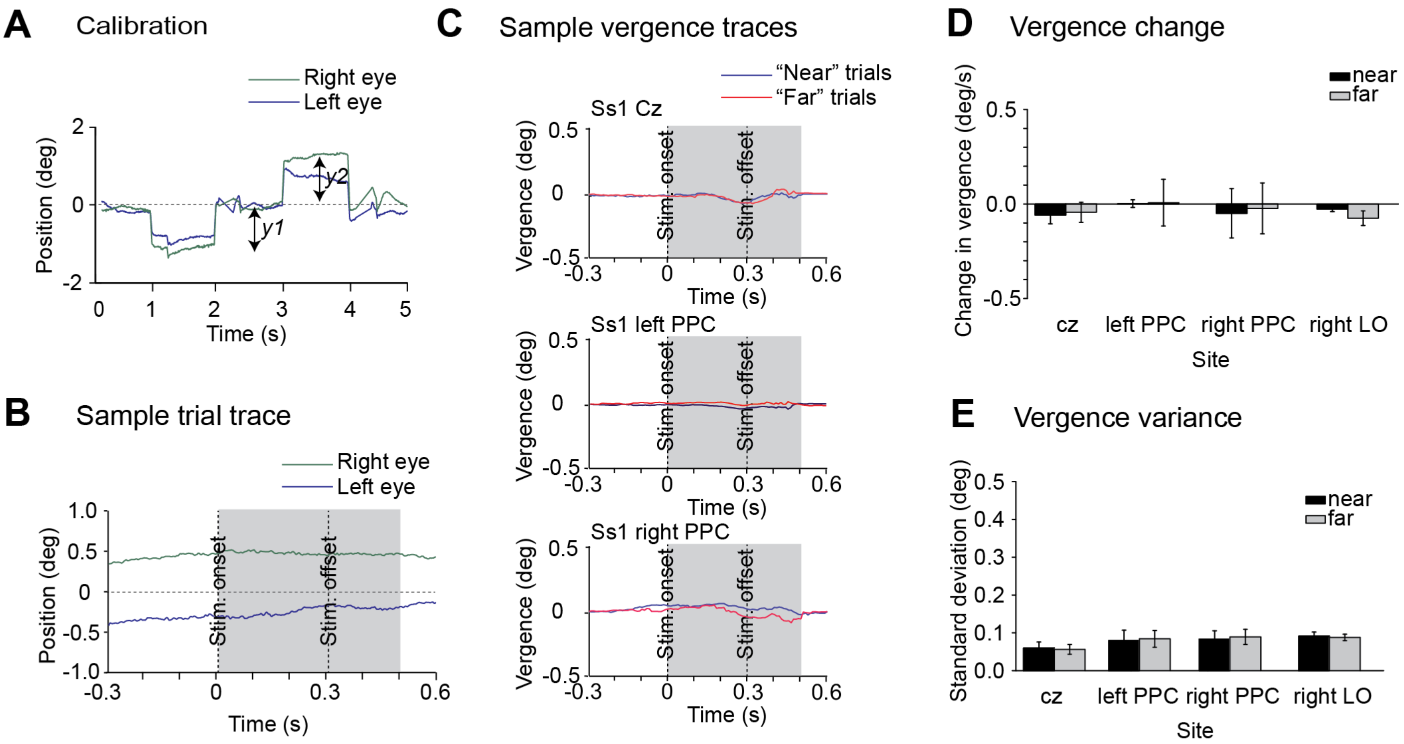


Eye-movement data presented for Cz, left- and right- PPC were measured pre-training, and eye-movement data presented for right LO were measure post-training, corresponding to the critical periods during which we observed task performance changes with TMS.

**(A)** Horizontal eye position data from a sample calibration window from one observer. Calibration data from each run were used to correct drift from centre (as indicated by the dashed line), and to compute a gain parameter corresponding to (y1 + y2) / 2. Data from each window of trials that followed each calibration block were recalibrated using these parameters.

**(B)** Horizontal eye position data from a sample rTMS trial from one observer. The portion of trial during which rTMS pulses were delivered is shown by the gray box. Stimulus onset and offset are indicated by the vertical dashed lines.

**(C)** Mean event-related horizontal vergence trace from a sample run from one observer. The interval of 300 ms prior to stimulus onset to 300 ms after stimulus offset is shown. Horizontal vergence angle was computed as the right horizontal eye minus the left horizontal eye. Thus, negative values for vergence correspond to positions that are nearer than the screen. The data are shown separately for near *vs*. far trials across stimulation sites. The portion of this window during which rTMS pulses were delivered is shown by the gray box. Stimulus onset and offset are indicated by the vertical dashed lines.

**(D)** To quantify any change of vergence that might take place during a trial, we fit a line to the eye vergence data of individual trials during the 300 ms window corresponding to stimulus presentation. We thereby quantified vergence changes on each trial in terms of the gradient ($\beta$) of the best fitting (least-squares) linear model to the data. Thereafter we compared the gradient terms for TMS vs. no TMS, finding no differences. In addition, during trials with rTMS stimulation, vergence did not vary depending on trial type (near/far), $F_{1,3}<1$, $p>.5$, or, stimulation site, $F_{3,6}<1$, $p>.5$. These data suggest that changes in vergence do not provide an account for the rTMS effects observed in our experiments.

(**E**) As a complementary analysis to **D**, we also computed the variance (standard deviation) in vergence position during the 300 ms window corresponding to stimulus presentation for each trial. We observed no differences in vergence variability between trials during which rTMS stimulations were applied and trials during which no stimulation was applied. Additionally, there were no differential effects of trial type (near/far), $F_{1,3}<1$, $p=.45$, or stimulation site, $F_{3,6}=1.2$, $p=.38$ on vergence variability.

## Fig. S4: Response times (Main Fig. 4)

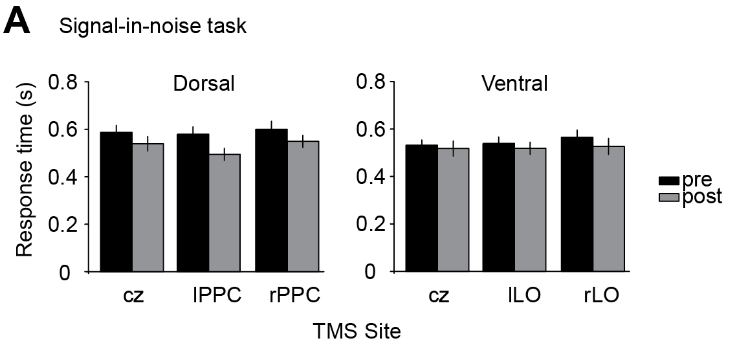


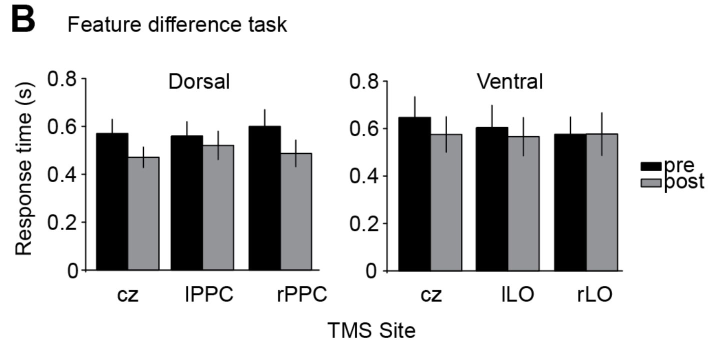


We analysed response time data from participants stimulated over both dorsal and ventral cortex, before and after training for both the (**a**) signal-in-noise and (**b**) feature difference tasks. Response times for the signal-in-noise task improved after training, $F_{1,22}$=6.02, $p$=.023, but did not differ between region of interest (PPC, dorsal *vs*. LO, ventral) ($F_{1,22}$<1, $p$=.48), or site (Cz, left, right) ($F_{2,44}$=2.71, $p$=.078), nor were there any significant interactions. Response times for the feature difference task improved after training ($F_{1,7}$=7.29, $p$=.031), but did not differ between region of interest (PPC, dorsal *vs*. LO, ventral), ($F_{1,7}$<1, $p$=.99) or site (Cz, left, right) ($F_{2,14}$<1, $p$=.89), nor were any interactions significant. These data suggest that there is little systematic effect of TMS over the sites of interest for response times on this task. This is expected as participants were instructed to perform the near threshold task accurately, and speed of response was not emphasised. Response times differ from the effects on threshold, thus do not appear to provide any form of explanation for the main experimental data reported in the paper.

## Table S1: Mean and SEM of the Talaraich coordinates for the stimulation sites of interest:

| | | Left hemisphere | | | Right hemisphere | | |
|---|---|---|---|---|---|---|---|
| **Region** | **N** | **x** | **y** | **z** | **x** | **y** | **z** |
| PPC | 9 | –34.1 (2.3) | –62.3 (4.5) | 42.3 (2.3) | 29 (1.6) | –66.5 (3.2) | 43.3 (1.7) |
| hMT+ | 6 | –44.6 (1.7) | –64.4 (1.1) | 6.6 (2.2) | 42.7 (1.2) | –57.7 (1.3) | 2.0 (1.3) |
| LO | 18 | –40.9 (0.6) | –68.3 (1.0) | –4.1 (1.0) | 41.5 (0.5) | –65.0 (1.0) | –3.7 (1.0) |

## Supplemental Experimental Procedures

### *Participants*

Participants (n=62) age ranged from 18 to 32 years (mean=22). All had normal or corrected-to-normal vision, were screened for stereo deficits, epilepsy or other neurological disorders in themselves or in their family, and provided written informed consent in line with local ethical review and approval of the work.

### *Stimuli*

Stimuli were random dot stereograms (RDS) (**Fig. 1**) surrounded by a grid of background squares (size = 0.5 deg), designed to provide a background reference and promote stable vergence. The RDS depicted a central target (diameter = 6 deg) surrounded by an annulus ("the surround", diameter = 12 deg). Individual dots subtended 0.15 deg and there were 6 dots/deg$^2$. Participants judged the position (in front / behind) of the central target relative to the surround. Task difficulty varied in one of two ways: 1) Signal-in-noise task: the target plane had disparity ± 6 arcmin (crossed or uncrossed) and we varied the percentage of dots defining the target (signal) relative to noise dots that had a random disparity within ± 12 arcmin. 2) Feature difference task: the surround had a disparity of 12 arcmin (crossed or uncrossed) and we varied the disparity between the target and surround in fine steps. For initial parietal stimulations (n=12) stimuli were presented on a 22 inch ViewSonic VX2260WM LCD display viewed through red/green anaglyphs. All subsequent experiments (including replications of parietal effects: **Fig. S2a,b**) employed a haploscope in which the two eyes viewed separate 22 inch Samsung (2233) LCD displays through front-silvered mirrors. Viewing distance was 50cm. Graphics rendering and anti-aliasing was implemented by an nVidia Quadro 4000 graphics card to display 1280 x 1024 pixels at 60 Hz on each display. Stimulus duration was 0.3s.

### *rTMS*

Stimulations were applied using a 70 mm figure-of-eight coil connected to a MagStim Rapid2 stimulator (MagStim, Whitland, UK) over left and right PPC, left and right LO, and Cz. The position of the coil was identified based on the 10-20 EEG coordinate system (left PPC (P3), right PPC (P4)), shown previously to correspond to posterior IPS (see **Table S1**), or using the Brainsight (Rogue Research) TMS-MRI coregistration system (left and right LO, hMT+/V5). The lateral occipital complex (LOC), the human motion complex (hMT+/V5) and retinotopic visual areas were defined using standard procedures [S4]. The 10-20 EEG coordinate system was also used to localise Cz. For nine participants, we obtained high-resolution anatomical (1 mm) scans with cod liver oil capsules (500 mg) positioned at electrode positions P3 (left PPC) and P4 (right PPC) of the 10-20 EEG coordinate system (**Fig. 2**; **Table S1**).

For all stimulation sites, the coil was placed tangential to the head with the handle pointing posteriorly (for parietal and Cz stimulation) or superiorly (for LO stimulation and hMT+/V5). Online stimulation was given at 10 Hz (5 pulses synchronised with stimulus onset) with a fixed intensity of 60% of the stimulator's maximum output [S5] for all sites of interest. Comparing task performance under TMS across areas could be problematic if the efficacy of the TMS perturbation varies between areas (e.g. due to differences in the distance of the area from the skull and/or differences in skull/muscle thickness). However, in our case, we assessed TMS effects of two different tasks within the same area, as well as before vs. after training within the same area. We applied TMS at a level that is compatible with other published work in this field, and found that the same amount of TMS produced dissociable effects on our two tasks. This fixed stimulator intensity resulted in performance reductions in both PPC and LO, meaning that this protocol was sufficiently sensitive to detect neural effects in different areas before and after training. That is, the same amount of TMS had an effect in PPC before training, but no effect after training; in contrast to TMS to ventral cortex that was effective after-, but not before-, training. Further performance disruption was comparable ($t_{22}<1$, $p$=.497) for PPC TMS before training (18.76% ± 5.73 SEM) and LO TMS after training (24.92% ± 6.84 SEM). No participants reported phosphenes over any stimulation site. To prevent overheating, the TMS coil was replaced after each run. The TMS protocol may induce a mixture of effects time-locked to stimulus presentation and carry-over effects.

The rTMS experiment consisted of three phases: 1) Pre-training tests carried over three separate days, each testing a different stimulation site (e.g., left PPC, right PPC, Cz or lLO, rLO, Cz or rhMT+, lhMT +, Cz). Within each session, the participant was tested on two runs (208 trials in total) of each of the signal-in-noise and feature difference tasks. rTMS was only applied during one task (i.e., signal-in-noise or feature difference task), and this was always the second task performed to avoid TMS carryover effects. 2) Training on a task that comprised 21 runs (2184 trials) completed over three consecutive days. 3) Post-training tests carried over three separate days which were identical to those completed pre-training. The order of stimulation sites was counterbalanced across participants, but was fixed between pre- and post-training tests for each observer. For each test and training run, task difficulty was adjusted according to two interleaved staircases determining thresholds at the 82%-correct level. For each participant, the threshold for a given run was computed as the mean of the thresholds from each staircase.

### *fMRI*

Region of interest localizer imaging data for the participants were acquired at the Birmingham University Imaging Centre using a 3-tesla Philips MRI scanner with an eight-channel head coil. Blood oxygen level–

dependent signals were measured with an echo-planar sequence (TE 35 ms; TR 2000 ms; 2.5 × 2.5 × 3 mm, 32 slices). For each participant, we additionally acquired a high-resolution (1 mm) anatomical scan. fMRI data were analysed with BrainVoyager QX (BrainInnovation B.V.). For each participant, we transformed anatomical data into Talairach space. Functional data were preprocessed using three-dimensional motion correction, slice time correction, linear trend removal and high-pass filtering (three cycles per run cut-off). LO was defined as the set of contiguous voxels in the lateral occipitotemporal cortex that showed significantly stronger activation for intact than scrambled images, consistent with previous reports [S6].

*Eye recording and analysis*

Binocular eye movements were recorded using an Eyelink 1000 remote video tracker (SR Research), with sampling rate 500 Hz. The system has a stated accuracy of 0.25 deg and resolution of 0.01 deg RMS. The tracker viewed participants' eyes through the (infrared transmitting) cold mirrors of the stereoscope.

On each run, observers were instructed to maintain fixation on a square marker (0.5 deg on each side) with horizontal and vertical nonius lines (0.3 deg in length). This square marker was centred and present throughout the entire run, but shifted horizontally (e.g., centre, +1 deg horizontal, centre, –1 deg horizontal, centre) during each calibration block that occurred at the start of each run, and once every 10 trials thereafter (11 blocks per run). Each calibration block lasted 5 seconds during which the configuration of fixation shifts was selected randomly between [0 –1 0 1 0] or [0 1 0 –1 0], where zero indicates a centred fixation marker, and ±1 indicate 1 deg shifts to the horizontal right or left, respectively.

To analyse the eye movement data of a run, we first converted raw gaze positions to degrees of visual angle. The time series data were then preprocessed by removing any data that corresponded to periods of blinks (average 7% of a given run for both trials with and without TMS) or saccades (average 12% of a given run for both trials with and without TMS), as identified by the EyeLink inbuilt detection functions. We followed this with a manual inspection of the data to ensure there were no additional blinks or saccades that were not detected by these functions. Any periods during which tracking was lost in one or both eyes or during which data were excessively noisy (due to instability of the eyetracker in determining pupil-corneal reflections) were additionally discarded (<1% of a given run). All removal of data were performed "blind" to experimental conditions, and required the agreement of two of the authors.

The remaining data were then subject to drift and gain correction using data from the calibration blocks. For each calibration block, we computed average centre coordinates of the observer's gaze from periods in which the fixation marker was centred, and a gain parameter that corresponded to the average horizontal amplitude of the observer's gaze during shifts of the fixation marker, (y1 + y2)/2 (**Fig. S3a**). The parameters from each calibration block were then used to recalibrate positional data from the window of trials that preceded it. Next, the recalibrated data were segregated into trial windows that included the period from 300 ms prior to stimulus onset to 300 ms after stimulus offset. Horizontal vergence angle was computed as the right horizontal eye position minus the left horizontal eye position (in relation to fixating at the centre of the screen). Thus, negative vergence values correspond to positions that are nearer relative to fixation)**.** Vergence changes and variability were computed across the 300 ms trial window corresponding to stimulus presentation only (**Fig. S3 d, e**).

*Statistical Analyses*

Statistical analysis was conducted in SPSS (SPSS Inc, Chicago, Ill). We analysed raw threshold values using repeated-measures ANOVAs, and applied Greenhouse-Geiser correction where appropriate. We used bonferonni corrected *t*-tests for post-hoc analyses.

## Supplemental References


S1. Silvanto, J., Lavie, N., & Walsh, V. (2005). Double dissociation of V1 and V5/MT activity in visual awareness. *Cerebral Cortex,* 15(11), 1736–1741.

S2. Walsh, V., Ashbridge, E., & Cowey, A. (1998). Cortical plasticity in perceptual learning demonstrated by transcranial magnetic stimulation. *Neuropsychologia*, 36(4), 363–367.

S3. Chang, D. H. F., Kourtzi, Z., & Welchman, A. E. (2013). Mechanisms for extracting a signal from noise as revealed through the specificity and generality of task training. *Journal of Neuroscience*, 33(27), 10962–10971

S4. Preston, T. J., Kourtzi, Z., & Welchman, A. E. (2009). Adaptive estimation of three-dimensional structure in the human brain. *Journal of Neuroscience*, 29(6), 1688–1698.

S5. Mevorach, C., Humphreys, G. W., & Shalev, L. (2006). Opposite biases in salience-based selection for the left and right posterior parietal cortex. *Nature Neuroscience*, 9(6), 740–742.

S6. Kourtzi, Z., & Kanwisher, N. (2001). Representation of perceived object shape by the human lateral occipital complex. *Science*, 293(5534), 1506–1509.
