# Supplementary material for: Training Transfers the Limits on Perception from Parietal to Ventral Cortex
Source: Curr Biol. 2014 Oct 20;24(20):2445–50. doi: 10.1016/j.cub.2014.08.058 (PMC4204932; doi:10.1016/j.cub.2014.08.058)
Supplement: Document S2. Article plus Supplemental Information [file mmc2.pdf]

Current Biology 24, 2445–2450, October 20, 2014 ©2014 The Authors http://dx.doi.org/10.1016/j.cub.2014.08.058

Report

Training Transfers the Limits on Perception from Parietal to Ventral Cortex

Dorita H.F. Chang,[1,2] Carmel Mevorach,[2] Zoe Kourtzi,[2,3,4] and Andrew E. Welchman[2,3,4,*]
[1]McGill Vision Research, Department of Ophthalmology, McGill University, Montreal, QC H3A 1A1, Canada
[2]School of Psychology, University of Birmingham, Edgbaston, Birmingham B15 2TT, UK
[3]Laboratory for Neuro- and Psychophysiology, KU Leuven, O&N II Herestraat 49, Box 1021, 3000 Leuven, Belgium
[4]Department of Psychology, University of Cambridge, Downing Street, Cambridge CB2 3EB, UK

Summary

Visually guided behavior depends on (1) extracting and (2) discriminating signals from complex retinal inputs, and these perceptual skills improve with practice [1]. For instance, training on aerial reconnaissance facilitated World War II Allied military operations [2]; analysts pored over stereoscopic photographs, becoming expert at (1) segmenting pictures into meaningful items to break camouflage from (noisy) backgrounds, and (2) discriminating fine details to distinguish V-weapons from innocuous pylons. Training is understood to optimize neural circuits that process scene features (e.g., orientation) for particular purposes (e.g., judging position) [3–6]. Yet learning is most beneficial when it generalizes to other settings [7, 8] and is critical in recovery after adversity [9], challenging understanding of the circuitry involved. Here we used repetitive transcranial magnetic stimulation (rTMS) to infer the functional organization supporting learning generalization in the human brain. First, we show dissociable contributions of the posterior parietal cortex (PPC) versus lateral occipital (LO) circuits: extracting targets from noise is disrupted by PPC stimulation, in contrast to judging feature differences, which is affected by LO rTMS. Then, we demonstrate that training causes striking changes in this circuit: after feature training, identifying a target in noise is not disrupted by PPC stimulation but instead by LO stimulation. This indicates that training shifts the limits on perception from parietal to ventral brain regions and identifies a critical neural circuit for visual learning. We suggest that generalization is implemented by supplanting dynamic processing conducted in the PPC with specific feature templates stored in the ventral cortex.

Results

We sought to identify the cortical circuits critically involved in (1) extracting signals and (2) discriminating features, and thereafter to determine how training modifies these circuits. We targeted these perceptual processes using two tasks that rely on them differentially: (1) a signal-in-noise task that involves extracting a target masked by noise versus (2) a feature-difference task that involves judging fine differences. We were particularly interested in generalization between tasks that—according to theoretical models [1, 10]—results from the optimization of distinct processing related to (1) filtering nonrelevant items from displays and (2) reading out representations of trained features. Although considerable behavioral evidence supports this framework [11], its neural basis is uncertain, as work on the neural basis of perceptual learning has typically trained and tested on the same task and stimuli, meaning that the stratified processes supporting learning could not be separated. One exception [12] demonstrated that neural activity associated with signal-in-noise judgments became unlinked to perceptual performance following training on a feature-difference task; however, the neural circuits involved in posttraining generalization were not revealed.

Participants viewed a 3D display (Figure 1) and judged whether the central target was in front or behind the surrounding annulus. In the signal-in-noise task, we varied the proportion of dots defining the target plane relative to distracting dots with randomly chosen depths. In the feature-difference task, we titrated the disparity between the center and surround under noise-free presentation. We measured discrimination thresholds by adaptively controlling either the (1) signal-to-noise ratio or (2) disparity, thereby ensuring that task difficulty was equated between tasks (and before versus after training). We then used training across tasks to track changes in both perceptual performance and the neural substrates. Previous work demonstrated asymmetric transfer between tasks: training on a feature-difference task improves signal-in-noise task performance, but not vice versa [1, 7, 10, 13, 14]. We thus focused on training the feature-difference task that supports transfer.

To probe the neural circuits involved, we used repetitive transcranial magnetic stimulation (rTMS) to temporarily disrupt processing in candidate regions of interest. We were a priori interested in posterior parietal cortex (PPC) that is involved in top-down attentional selection of targets in noise [15] by means of figure-ground segmentation [16] and learning [17], in contrast to ventral areas that process disparity-defined forms [18, 19] and feature templates [20]. We therefore measured performance on (1) feature-difference and (2) signal-in-noise tasks while participants received rTMS over PPC (dorsal) or lateral occipital (LO) (ventral) areas (Figure 2; Table S1 available online). To control for generalized interference from rTMS, we stimulated a control site (Cz) to provide a baseline for psychophysical performance (Figure S1A shows raw thresholds).

Before considering the rTMS results, we confirmed the asymmetric transfer between tasks [7, 13] by considering participants' performance on both the trained and untrained tasks before and after 3 days of training on one of the tasks. Training on the feature-difference task (Figure 3A) improved performance on both the feature-difference task (Figure 3B; t_{26} = 8.79, $p < 0.001$) and the signal-in-noise task (Figure 3C; t_{26} = 9.18, $p < 0.001$). By contrast, training on the signal-in-noise task (Figure 3D) benefited this task (Figure 3E; t_5 = 3.67, p = 0.014) but there was no transfer to the feature-difference

*Correspondence: aew69@cam.ac.uk
This is an open access article under the CC BY license (http://creativecommons.org/licenses/by/3.0/).

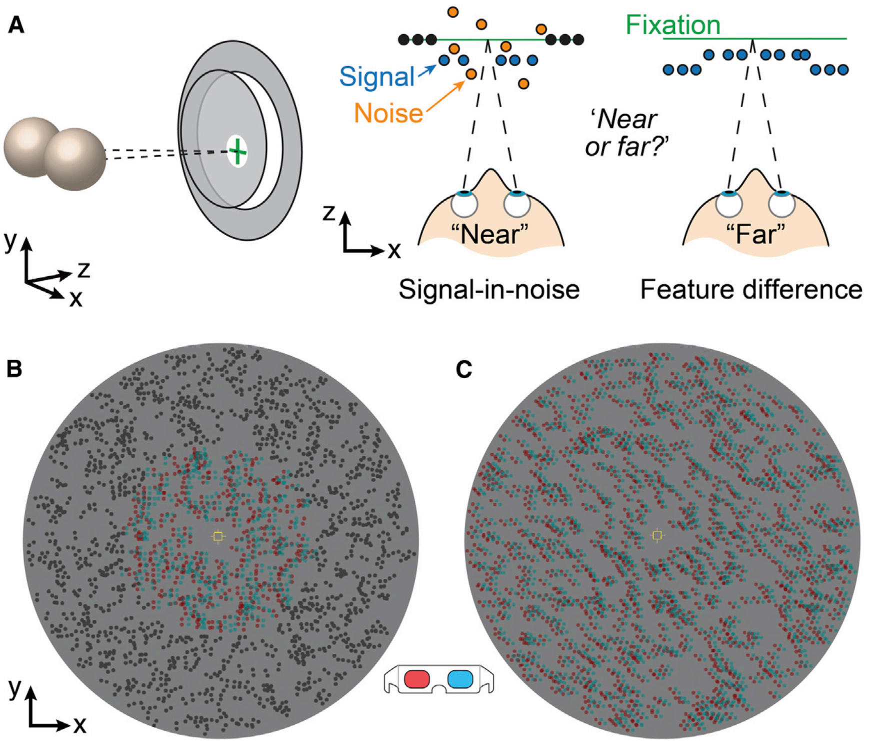

Figure 1. Stimuli for the Signal-in-Noise and Feature-Difference Tasks

(A) Cartoon illustrations of the stimuli: the two eyes view a center-surround display. Signal-in-noise task: the target disparity was fixed at ±6 arcmin, and we varied the proportion of target signal dots relative to noise dots with randomly chosen disparities within ±12 arcmin. Feature-difference task: the disparity difference between the center and surround (±12 arcmin) was varied in fine steps. For both tasks, the participant decided whether the center is nearer or farther than the surround.

(B and C) Sample random dot stimuli rendered as red-cyan anaglyphs for the signal-in-noise (B) and feature-difference tasks (C). The center was 6° in diameter, and the surround was 12° in diameter. Participants fixated on the small square marker at the center of the display.

task (Figure 3F; $t_5 < 1$, p = 0.55). This asymmetry was supported by an rANOVA with a significant interaction of session (pre versus post) and training task (feature-difference versus signal-in-noise) for the feature task ($F_{1,31}$ = 6.60, p = 0.015), but not the signal-in-noise task ($F_{1,31}$ = 2.52, p = 0.122).

Considering the results of rTMS before training, we found worse performance (i.e., higher thresholds) for left PPC rTMS than Cz, in contrast to LO stimulation, where performance was unaffected (Figure 4A). rANOVAs conducted on the raw discrimination thresholds indicated a significant difference between stimulation sites (Cz, left PPC, right PPC) for dorsal rTMS ($F_{1,11}$ = 9.79, p = 0.01), in contrast to no significant differences ($F_{2,22} < 1$, p = 0.915) between sites (Cz, left LO, right LO) for ventral stimulation. The effect in dorsal cortex was specific to the left PPC (t_{11} = 3.13, p = 0.009) and replicable (Figure S1C). This left lateralization was anticipated, because noise dots were distracting: damage to left parietal cortex impairs patients' abilities to ignore salient distracting information [21], whereas healthy adults are poorer at inhibiting high-salience distracters during TMS over left PPC [22].

We found contrasting results for the pretraining tests on the feature-difference task (Figure 4B). In particular, PPC stimulation did not affect judgments ($F_{2,10} < 1$, p = 0.446) but rTMS to LO did ($F_{2,10}$ = 9.18, p = 0.005). This LO effect was more pronounced in the right hemisphere (although not statistically significant). These dissociated results between dorsal and ventral areas for signal-in-noise and feature tasks suggest distinct contributions to perception: left parietal cortex may be critically involved in external noise filtering, whereas feature representations in ventral LO may support fine discriminations.

Following the pretraining sessions, we tested whether training on the feature-difference task caused changes in the neuronal circuits supporting perceptual judgments. Given that feature-difference training promotes transfer to the signal-in-noise task, it was of critical interest to determine the role of ventral circuits in the signal-in-noise task after training. In contrast to the pretraining results, we found that signal-in-noise task performance was unaffected by left (or right) PPC stimulation ($F_{2,22} < 1$, p = 0.869). Strikingly, performance was instead significantly ($F_{2,22}$ = 5.27, p = 0.01) worse under ventral stimulation (Figure 4C).

This reversal of rTMS-induced deficits for the signal-in-noise task from dorsal to ventral cortex was supported by a significant three-way interaction (rANOVA, $F_{2,44}$ = 6.40, p = 0.004) between training session (pre versus post), location (dorsal versus ventral), and stimulation site (left hemisphere, right hemisphere, Cz). Importantly, a significant interaction ($F_{1.6,35.2}$ = 9.06, p = 0.001) between session and location confirmed the dissociable role of these areas in the signal-in-noise task before versus after training. A follow-up two-way rANOVA showed a significant interaction of training and site (left PPC, right PPC, Cz) ($F_{2,22}$ = 9.76, p = 0.001), consistent with decreased performance before, but not after, training for left PPC. For ventral rTMS, a significant interaction of training and site (left LO, right LO, Cz) ($F_{2,22}$ = 6.29, p = 0.007) was also observed, but the pattern was reversed: performance under ventral stimulation decreased after, but not before, training. The effect in ventral cortex for the signal-in-noise task after training was stronger in the right hemisphere (t_{11} = 2.23, p = 0.048), echoing the pretraining results for performance on the feature-difference task.

These dissociable effects of rTMS suggest a fundamental change in the cortical areas that limit performance on the signal-in-noise task, such that there is a decreased contribution of parietal cortex and an increased role of LO after training. Further testing revealed that ventral sites remained important for the feature-difference task: LO stimulation after training remained disruptive for feature-difference judgments (Figure 4D; main effect of stimulation site: $F_{2,10}$ = 9.18, p = 0.005, but no interaction with training: $F_{2,10} < 1$, p = 0.901). In common with the preceding results, rTMS effects were stronger in right LO (t_5 = 2.61, p = 0.047).

We next asked whether changes in the circuit involved in signal-in-noise identification depend on training on the feature-difference task. We first tested participants (n = 8) on the signal-in-noise task before and after 3 days of rest

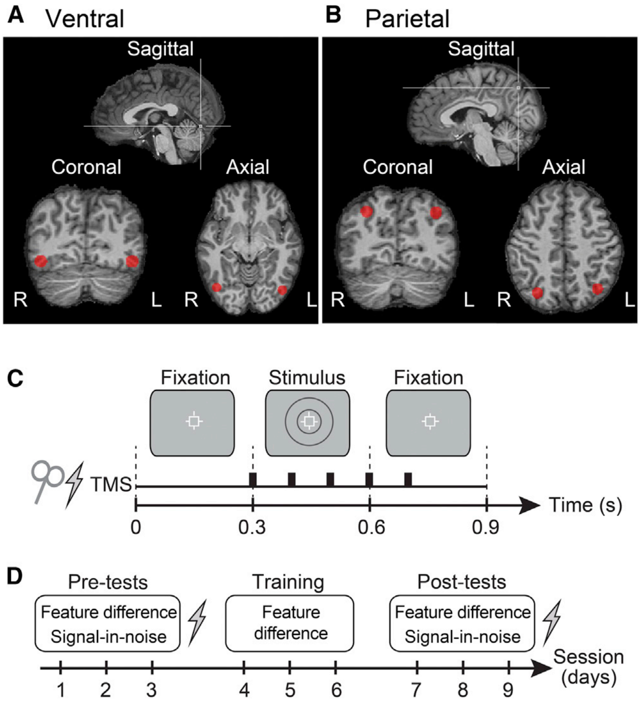

Figure 2. Stimulation Sites and Experimental Design

(A and B) Anatomical locations of ventral (A) and parietal (B) stimulation sites. LO was defined based on functional magnetic resonance imaging (fMRI) activations; left and right PPC were identified using MRI scans with cod liver oil capsules positioned at P3 and P4 of the 10–20 electroencephalography coordinate system.

(C) Stimulus presentation and TMS timeline. Online stimulation was given at 10 Hz (five pulses synchronized with stimulus onset) with a fixed intensity of 60% of the stimulator's maximum output.

(D) Experimental protocol: pretraining TMS tests (3 days), training (3 days), and posttraining TMS tests (3 days). During pre- and posttesting sessions, participants performed both tasks, but rTMS was delivered during only one of the tasks. The order of stimulation sites was counterbalanced across participants, but was fixed between pre- and posttraining tests for each observer. For each test and training run, task difficulty was adjusted by varying the stimulus according to two interleaved staircases determining thresholds at the 82%-correct level.

See also Figure S2.

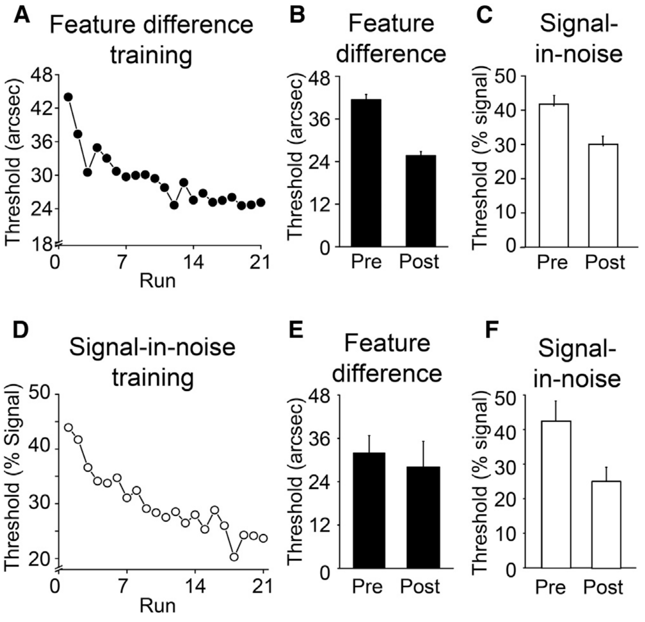

Figure 3. Behavioral Thresholds before and after Training

(A) Threshold changes across the 21 training runs (2,184 trials) of the feature-difference task.

(B and C) Mean performance for the feature-difference and signal-in-noise discrimination tasks, before and after training, pooled across participants (n = 27).

(D) Threshold changes across the 21 training runs (2,184 trials) of the signal-in-noise task.

(E and F) Mean performance for the feature-difference and signal-in-noise discrimination tasks, before and after training, pooled across participants (n = 6). Error bars represent ±1 SEM. (Note that mean pretraining thresholds for participants in B are slightly higher than for the participants in E; however, selecting participants from the feature-difference training group, B, with comparable pretraining thresholds to the signal-in-noise training group, E, revealed a clear training effect, suggesting that the lack of significant transfer for participants trained on the signal-in-noise task could not be ascribed to a floor effect whereby it was not possible for thresholds to improve further.)

(Figure S2A). We found that parietal stimulation remained disruptive when participants were not actively trained: there was a main effect ($F_{1,7}$ = 27.09, p = 0.001) of site (left PPC, Cz) but no interaction with session ($F_{1,7} < 1$, p = 0.664). Second, we trained new participants (n = 6) on the signal-in-noise task rather than the feature-difference task (Figure S2B). We found that ventral stimulation had no effect on signal-in-noise task performance (before or) after training on this task ($F_{1,5} < 1$, p = 0.84), indicating that feature-difference training was critical. This could not be due to insufficient training for the signal-in-noise task, because learning rates were matched (Figure S2C) [13]. We speculate that after signal-in-noise training, information in earlier visual areas may be critical, because the high spatial resolution of earlier sensory neurons affords refined signal-in-noise discrimination following coarser target detection at higher processing stages [23]. Third, we retested available participants trained on the feature-difference task 1–6 months after initial testing. We found that shifts in the cortical loci limiting signal-in-noise judgments lasted for a long period: ventral (rather than dorsal) stimulation retained its disruptive effect for each individual participant (Figure S1D). Taken together, these results suggest that training on feature differences changes the functional contributions of dorsal and ventral cortex for perceptual judgments in noisy displays. This functional reweighting of the circuit involved in target identification from noise is specific and longer term in nature, requiring training on a task designed to boost feature templates.

To make a direct comparison between tasks measured in different units, we computed percent change in threshold before versus after training (although note that this approach is not without complication [13, 24]). We found a significant interaction between location, task, and rTMS site ($F_{1,29}$ = 4.39, p = 0.045), highlighting dissociable effects between tasks before versus after training. This dissociated pattern of results made experimental artifacts unlikely. First, nonspecific improvements in task performance could not explain our findings, because generalization was asymmetric. Second, the adaptive psychophysical procedure ensured that difficulty was equated for the different tasks before and after training, ruling out explanations based on general attentional demands. Third, any differences in rTMS efficacy between sites could not account for differences before versus after training. Further,

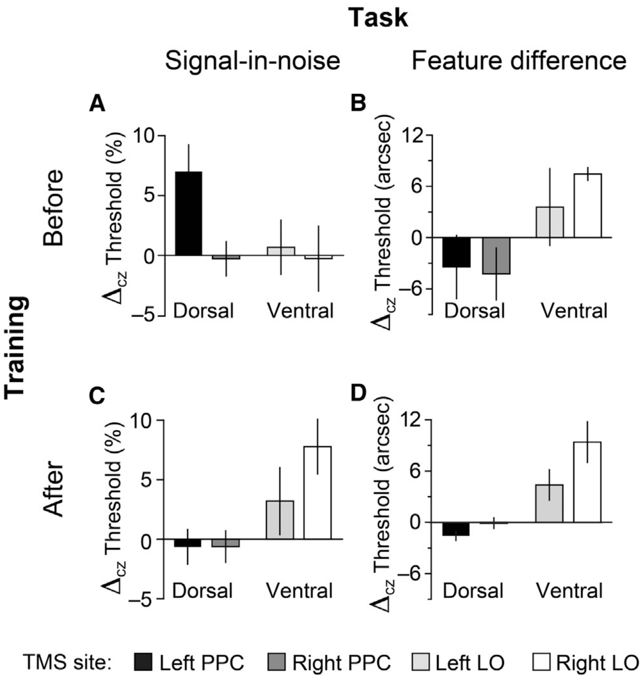

Figure 4. The Effects of Dorsal and Ventral rTMS on Signal-in-Noise and Feature-Difference Task Performance before versus after Training

(A) Mean performance (relative to Cz baseline) for the signal-in-noise task before training with rTMS over dorsal (n = 12) or ventral (n = 12) areas; see Figure S1 for unnormalized thresholds.

(B) Mean performance (relative to Cz baseline) for the feature-difference task before training with rTMS over dorsal (n = 6) or ventral (n = 6) areas.

(C) Mean performance for the signal-in-noise task after training with rTMS over dorsal or ventral areas.

(D) Mean performance for the feature-difference task after training with rTMS over dorsal or ventral areas.

Error bars represent ±1 SEM. See also Figures S1, S3, and S4.

performance disruption was comparable for PPC TMS before training and LO TMS after training. Fourth, we tested whether rTMS to dorsal versus ventral areas during pretraining sessions might interfere differentially with participants' ability to learn on subsequent days. We found no differences in the total learned ($F_{2,28} < 1$, p = 0.48) or learning rate ($F_{2,28} < 1$, p = 0.43) for participants who received no stimulation or rTMS of different sites (Figure S2D). Fifth, measuring binocular eye movements during rTMS showed that stimulation did not disrupt eye movement control (critical for stereopsis): eye vergence was stable and not systematically affected by rTMS (Figure S3), consistent with a previous report [25]. Finally, we analyzed participants' response times (Figure S4); these quickened following training (signal-in-noise task, $F_{1,22}$ = 6.02, p = 0.023; feature-difference task, $F_{1,7}$ = 7.29, p = 0.031) but did not differ between sites, as expected for threshold measurement tasks where participants were instructed to be as accurate as possible.

Discussion

Here we provide evidence for functional reweighting of a circuit that supports perceptual judgments through training. We tested performance on two tasks that differentiate the stages optimized during perceptual learning [1, 10]. This allowed us to identify cortical loci that limit performance on (1) signal extraction and noise filtering versus (2) the representation of features. These fundamental perceptual abilities critically depend on the dynamic processing capacities of the PPC versus template storing in ventral cortex. Thereafter, we showed that training designed to boost feature templates changes the loci that limit task performance: the signal-in-noise task is no longer critically limited by parietal activity but rather by ventral cortex. This identifies a cortical basis for theoretical models of learning that posit that feature-difference training optimizes the readout of feature templates [1, 10, 26].

Previous electrophysiological [27–29] and neuroimaging studies [30–33] focused on changes in neural responses for a given task and stimulus set following dedicated training on that task and stimulus set. However, this does not allow the computational stages involved to be differentiated, because optimization could take place at multiple levels. Here we took the approach of contrasting two tasks that rely differentially (but not exclusively) on (1) signal extraction and (2) feature discrimination. By examining how training affects performance not only on the trained task but also on a different untrained one, we uncover the cortical basis of the hypothesized mechanisms [7, 13]. We propose that performance in both the signal-in-noise task and the feature-difference task engages parietal and ventral loci but that the extent of activation differs. An observer's judgment can be no better than the noisiest estimation stage. For the signal-in-noise task, parietal cortex initially imposes this limit on performance. However, following training, readout weights are optimized [10]; in consequence, feature representations in ventral cortex become the limiting stage that determines task performance.

Previously, Chowdhury and DeAngelis [12] showed that generalized training from a feature task reduced the involvement of MT/V5 on a signal-in-noise task: reversible inactivation disrupted the monkey's perceptual judgments before, but not after, training. Similarly, TMS over human PPC can produce perceptual interference before, but not after, training [34], suggesting that the effects of dorsal stimulation can diminish following training. Our results support this idea; however, this work did not identify the loci responsible for posttraining performance. Critically, we demonstrate that ventral circuits support signal-in-noise task performance after training, indicating that learning changes the limits on visual perception from the posterior parietal to the ventral cortex. We assessed the involvement of hMT+/V5 under our paradigm, testing new observers (n = 6) on the signal-in-noise task before and after training on the feature-difference task. We found no interference on task performance from rTMS before or after training (Figure S1E). This difference from the macaque likely reflects the absence of motion from our stimuli.

We conjecture that training on fine differences optimizes the representations of disparity features in LO, consistent with evidence for disparity processing in macaque inferotemporal cortex [18, 19] and downstream V4 [35]. These boosted features facilitate figure-ground segmentation and the identification of targets in noise, diminishing the need for filtering by the parietal cortex. This process may involve augmented Hebbian reweighting, where a single set of readout weights is modified through training on feature differences [36]. Under this view, our data point to LO as the locus for these readout weights. The fact that rTMS was slightly stronger in rLO is compatible with evidence that right temporal cortex has a better capacity for template representations [37] and that right ventral TMS affects judgments of object properties [38, 39]. The maintained

role of this area for feature discriminations before and after training suggests that it plays a key role in depth-perception tasks. Finally, it is likely that the cortical network involved in perceptual learning extends beyond the areas we targeted, depending on the tasks and stimuli used. Nevertheless, our findings indicate a key type of circuit reweighting for generalization through stored representations that may be applicable to other stimuli and tasks.

The brain retains considerable capacity for plasticity in adulthood. Our finding of a functional dissociation between the dorsal and ventral regions before and after training highlights changes in the functional roles of regions underlying perception. The changes we observe may represent the operation of a general processing strategy through which the brain stores information from previous experience in ventral circuits to reduce the need for dynamic processing by the dorsal stream. Thus, task generalization may paradoxically depend on bolstering specific feature representations stored in the ventral cortex. As such, there may be value in boosting feature representations to ameliorate healthy (e.g., aging [40]) and clinical (e.g., attention-deficit/hyperactivity disorder [41]; neuropsychological patients [21]) populations who show impaired ability to ignore distracting information during everyday tasks.

Supplemental Information

Supplemental Information includes Supplemental Experimental Procedures, four figures, and one table and can be found with this article online at http://dx.doi.org/10.1016/j.cub.2014.08.058.

Acknowledgments

We thank M. Dexter for technical support and C. Chambers, A. Seitz, and R. Fleming for comments. The research received funding from the European Community's Seventh Framework Programme (FP7/2007–2013, PITN-GA-2008-214728, and PIIF-GA-2011-299610) and the Wellcome Trust (095183/Z/10/Z).

Received: June 16, 2014
Revised: July 18, 2014
Accepted: August 22, 2014
Published: October 2, 2014

References

1. Dosher, B.A., and Lu, Z.L. (1998). Perceptual learning reflects external noise filtering and internal noise reduction through channel reweighting. Proc. Natl. Acad. Sci. USA *95*, 13988–13993.
2. Downing, T. (2011). Spies in the Sky (London: Hachette UK).
3. Gilbert, C.D., Sigman, M., and Crist, R.E. (2001). The neural basis of perceptual learning. Neuron *31*, 681–697.
4. Poggio, T., Fahle, M., and Edelman, S. (1992). Fast perceptual learning in visual hyperacuity. Science *256*, 1018–1021.
5. Karni, A., and Sagi, D. (1991). Where practice makes perfect in texture discrimination: evidence for primary visual cortex plasticity. Proc. Natl. Acad. Sci. USA *88*, 4966–4970.
6. Schoups, A.A., Vogels, R., and Orban, G.A. (1995). Human perceptual learning in identifying the oblique orientation: retinotopy, orientation specificity and monocularity. J. Physiol. *483*, 797–810.
7. Dosher, B.A., and Lu, Z.L. (2005). Perceptual learning in clear displays optimizes perceptual expertise: learning the limiting process. Proc. Natl. Acad. Sci. USA *102*, 5286–5290.
8. Xiao, L.-Q., Zhang, J.-Y., Wang, R., Klein, S.A., Levi, D.M., and Yu, C. (2008). Complete transfer of perceptual learning across retinal locations enabled by double training. Curr. Biol. *18*, 1922–1926.
9. Ding, J., and Levi, D.M. (2011). Recovery of stereopsis through perceptual learning in human adults with abnormal binocular vision. Proc. Natl. Acad. Sci. USA *108*, E733–E741.
10. Dosher, B.A., Jeter, P., Liu, J., and Lu, Z.-L. (2013). An integrated reweighting theory of perceptual learning. Proc. Natl. Acad. Sci. USA *110*, 13678–13683.
11. Lu, Z.-L., Hua, T., Huang, C.-B., Zhou, Y., and Dosher, B.A. (2011). Visual perceptual learning. Neurobiol. Learn. Mem. *95*, 145–151.
12. Chowdhury, S.A., and DeAngelis, G.C. (2008). Fine discrimination training alters the causal contribution of macaque area MT to depth perception. Neuron *60*, 367–377.
13. Chang, D.H.F., Kourtzi, Z., and Welchman, A.E. (2013). Mechanisms for extracting a signal from noise as revealed through the specificity and generality of task training. J. Neurosci. *33*, 10962–10971.
14. Huang, X., Lu, H., Tjan, B.S., Zhou, Y., and Liu, Z. (2007). Motion perceptual learning: when only task-relevant information is learned. J. Vis. *7*, Article 14.
15. Corbetta, M., and Shulman, G.L. (2002). Control of goal-directed and stimulus-driven attention in the brain. Nat. Rev. Neurosci. *3*, 201–215.
16. Roelfsema, P.R., and van Ooyen, A. (2005). Attention-gated reinforcement learning of internal representations for classification. Neural Comput. *17*, 2176–2214.
17. Law, C.-T., and Gold, J.I. (2008). Neural correlates of perceptual learning in a sensory-motor, but not a sensory, cortical area. Nat. Neurosci. *11*, 505–513.
18. Uka, T., Tanabe, S., Watanabe, M., and Fujita, I. (2005). Neural correlates of fine depth discrimination in monkey inferior temporal cortex. J. Neurosci. *25*, 10796–10802.
19. Janssen, P., Vogels, R., and Orban, G.A. (2000). Selectivity for 3D shape that reveals distinct areas within macaque inferior temporal cortex. Science *288*, 2054–2056.
20. Kuai, S.-G., Levi, D., and Kourtzi, Z. (2013). Learning optimizes decision templates in the human visual cortex. Curr. Biol. *23*, 1799–1804.
21. Mevorach, C., Humphreys, G.W., and Shalev, L. (2006). Effects of saliency, not global dominance, in patients with left parietal damage. Neuropsychologia *44*, 307–319.
22. Mevorach, C., Humphreys, G.W., and Shalev, L. (2006). Opposite biases in salience-based selection for the left and right posterior parietal cortex. Nat. Neurosci. *9*, 740–742.
23. Ahissar, M., and Hochstein, S. (2004). The reverse hierarchy theory of visual perceptual learning. Trends Cogn. Sci. *8*, 457–464.
24. Fine, I., and Jacobs, R.A. (2002). Comparing perceptual learning tasks: a review. J. Vis. *2*, 190–203.
25. Kapoula, Z., Isotalo, E., Müri, R.M., Bucci, M.P., and Rivaud-Péchoux, S. (2001). Effects of transcranial magnetic stimulation of the posterior parietal cortex on saccades and vergence. Neuroreport *12*, 4041–4046.
26. Li, R.W., Levi, D.M., and Klein, S.A. (2004). Perceptual learning improves efficiency by re-tuning the decision 'template' for position discrimination. Nat. Neurosci. *7*, 178–183.
27. Li, W., Piëch, V., and Gilbert, C.D. (2008). Learning to link visual contours. Neuron *57*, 442–451.
28. Ghose, G.M., Yang, T., and Maunsell, J.H.R. (2002). Physiological correlates of perceptual learning in monkey V1 and V2. J. Neurophysiol. *87*, 1867–1888.
29. Schoups, A., Vogels, R., Qian, N., and Orban, G. (2001). Practising orientation identification improves orientation coding in V1 neurons. Nature *412*, 549–553.
30. Yotsumoto, Y., Watanabe, T., and Sasaki, Y. (2008). Different dynamics of performance and brain activation in the time course of perceptual learning. Neuron *57*, 827–833.
31. Shibata, K., Watanabe, T., Sasaki, Y., and Kawato, M. (2011). Perceptual learning incepted by decoded fMRI neurofeedback without stimulus presentation. Science *334*, 1413–1415.
32. Schwartz, S., Maquet, P., and Frith, C. (2002). Neural correlates of perceptual learning: a functional MRI study of visual texture discrimination. Proc. Natl. Acad. Sci. USA *99*, 17137–17142.
33. Furmanski, C.S., Schluppeck, D., and Engel, S.A. (2004). Learning strengthens the response of primary visual cortex to simple patterns. Curr. Biol. *14*, 573–578.
34. Walsh, V., Ashbridge, E., and Cowey, A. (1998). Cortical plasticity in perceptual learning demonstrated by transcranial magnetic stimulation. Neuropsychologia *36*, 363–367.
35. Shiozaki, H.M., Tanabe, S., Doi, T., and Fujita, I. (2012). Neural activity in cortical area V4 underlies fine disparity discrimination. J. Neurosci. *32*, 3830–3841.

36. Lu, Z.-L., Liu, J., and Dosher, B.A. (2010). Modeling mechanisms of perceptual learning with augmented Hebbian re-weighting. Vision Res. *50*, 375–390.
37. Levy, J., Trevarthen, C., and Sperry, R.W. (1972). Reception of bilateral chimeric figures following hemispheric deconnexion. Brain *95*, 61–78.
38. Pitcher, D., Charles, L., Devlin, J.T., Walsh, V., and Duchaine, B. (2009). Triple dissociation of faces, bodies, and objects in extrastriate cortex. Curr. Biol. *19*, 319–324.
39. Ellison, A., and Cowey, A. (2006). TMS can reveal contrasting functions of the dorsal and ventral visual processing streams. Exp. Brain Res. *175*, 618–625.
40. Kuai, S.-G., and Kourtzi, Z. (2013). Learning to see, but not discriminate, visual forms is impaired in aging. Psychol. Sci. *24*, 412–422.
41. Castellanos, F.X., and Tannock, R. (2002). Neuroscience of attention-deficit/hyperactivity disorder: the search for endophenotypes. Nat. Rev. Neurosci. *3*, 617–628.

Current Biology, Volume *24*

Supplemental Information

Training Transfers the Limits on Perception from Parietal to Ventral Cortex

Dorita H.F. Chang, Carmel Mevorach, Zoe Kourtzi, and Andrew E. Welchman

Fig S1: Effects of parietal rTMS, retention of rLO effect, and hMT+/V5 stimulation (Fig. 4)

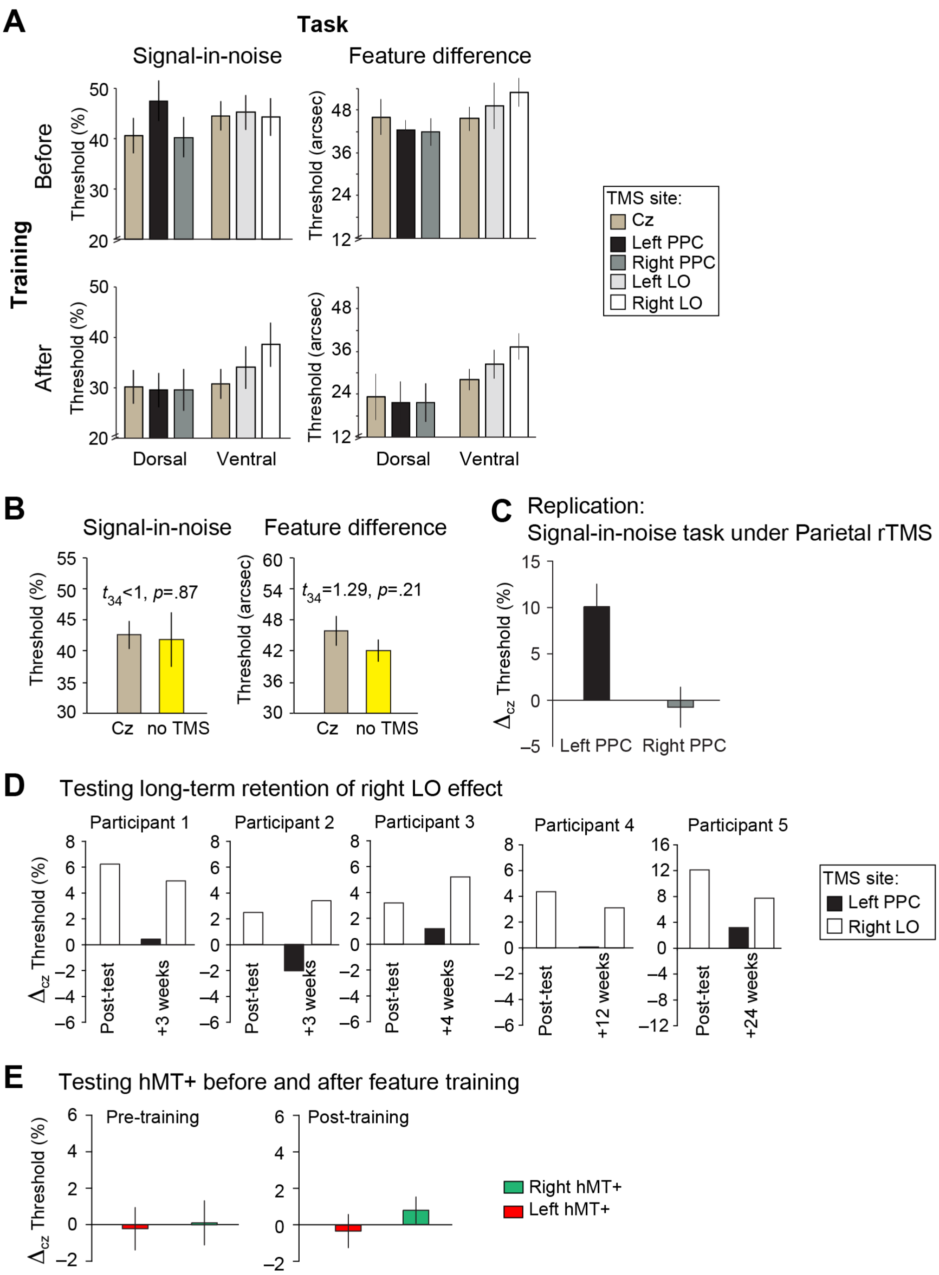

(**A**) Between-subjects average thresholds for the signal-in-noise and feature tasks during rTMS of the control (Cz) or regions of interest (PPC or LO) sites, before- and after- training. These data are presented relative to the Cz thresholds in Main Figure 4. The error bars depict the s.e.m.

(**B**) Comparison between control site (Cz) *vs*. no rTMS. Between-subjects mean thresholds are shown (with error bars for the s.e.m.). Thresholds were similar under control site TMS and when no TMS was applied.

(**C**) Replication of the parietal rTMS effect. We repeated our assessment of the effects of left PPC and right PPC stimulation (relative to Cz) during performance on the signal-in-noise (n=9). As in the main experiment, we observed a significant difference between stimulation over the different sites ($F_{2,16}$=12.7, p<.001), with pronounced threshold increases under left PPC stimulation.

(**D**) We investigated the long-term effects of training by recalling five observers at 3-4 weeks (Participants 1-3), 12 weeks (participant 4), or 24 weeks (Participant 5) following training. We retested performance on the signal-in-noise task during stimulation over left PPC, right LO and Cz (control). No additional task training was provided before these tests. For all five observers, stimulation of right LO still impaired performance, indicating long-term changes in the importance of ventral circuits.

(**E**) We tested new observers (n=6) on the signal-in-noise task with rTMS over right and left hMT+, and Cz, before and after training on the feature difference task. Thresholds improved after training, but were not differentially affected by hMT+/V5 stimulations versus Cz [rANOVA on site (right and left hMT+, Cz) and test (pre-, post-training); main effect of training, $F_{1,5}$=57.3, p<.001, but no other significant effects]. Previous work using 60% intensity stimulation affected perceptual judgments [S1], suggesting that it is unlikely that the null effect for hMT+/V5 stimulation was due to insufficient rTMS intensity.

Fig. S2: Considerations of the role of different types of training (Main Fig.3)

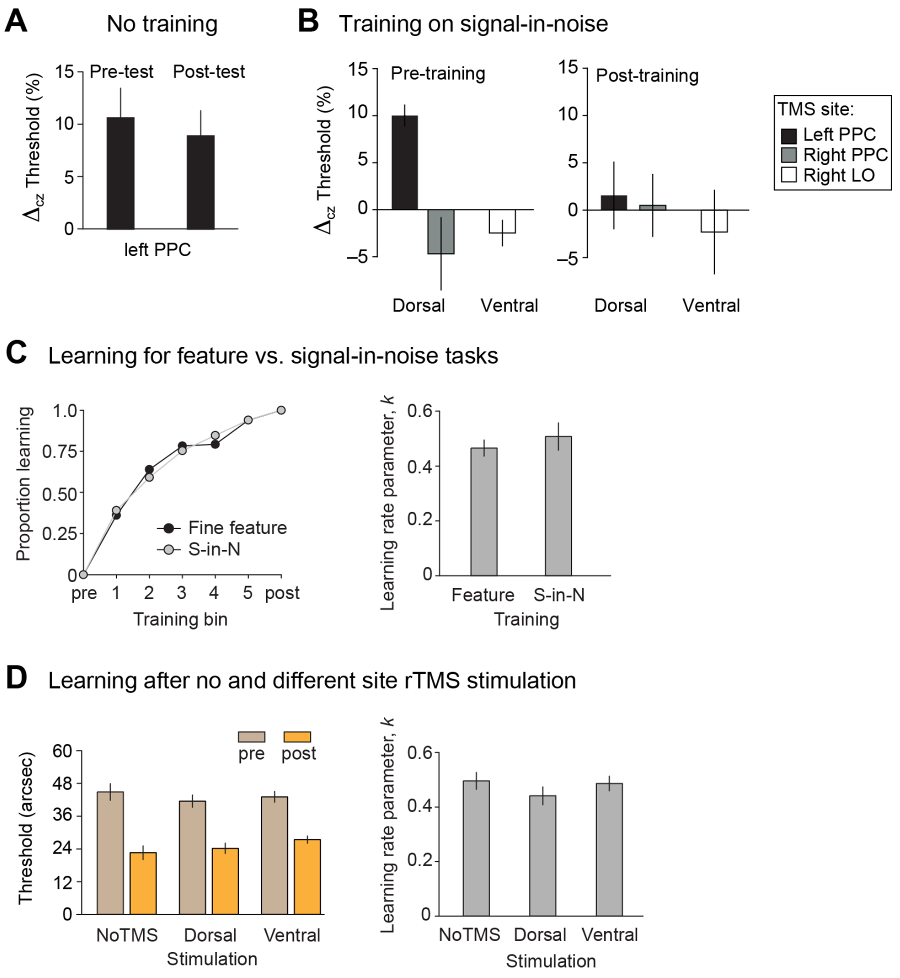

(**A**) We tested new observers (n=8) on the signal-in-noise task with rTMS over left PPC and Cz before and after three days of rest. Stimulations over left PPC affected thresholds significantly relative to baseline Cz both during initial tests (t_7=3.69, p=.008) and re-tests (t_7=3.62, p=.008). Additionally, thresholds relative to Cz did not differ between tests (t_7<1, p=.66) indicating that active training was required to see the reduction in the contribution of left parietal cortex to performance on the signal-in-noise task.

(**B**) We tested a new group of observers (n=6) on the signal-in-noise task with stimulation over Cz, left PPC, right PPC and right LO before and after three consecutive days of training on the signal-in-noise task (rather than the feature difference task which was used in training for the main experiments). We found a significant training by stimulation site interaction ($F_{3,15}$=4.62, p=.018) which was due to significantly worse performance for left PPC stimulation before training ($F_{3,15}$=6.5, p=.005) but not afterwards ($F_{3,15}$<1, p=.63). These data demonstrate that the perceptual contribution of the posterior parietal cortex is affected by training [S2]. Critically however, increasing the role of ventral area LO is not a general feature caused by training: without training on fine feature differences, we do not see a role for ventral areas. This suggests that training paradigms that boost feature representations may be necessary for re-weighting and transfer.

(**C**) We compared behavioural improvements for the tasks by considering the normalised learning functions for training on feature differences and the signal-in-noise task. As the tasks measure thresholds in different units—disparity vs. signal to noise proportion—we calculated normalised performance in each session as a proportion of the total amount learnt over the course of training – thus the y-axis is normalised between zero (pre-training) and one (post-training) performance. The graph depicts the mean learning function across individuals (Feature task n=24; signal-in-noise task n=6). Consistent with behavioural data presented elsewhere [S3] we found very similar learning functions for the feature difference and signal-in-noise tasks. Formally, we fit each individual's learning function with an exponential saturating learning model with the form $b = k \ln(a)$, where a is the training block, b is the proportion of learning and k is the fitted learning rate parameter. We found no reliable difference in the learning rate parameter for participants trained on the feature task vs. the signal-in-noise task (t_{28}<1, p=.40) indicating similar levels of improvement for the two tasks.

(**D**) We tested whether rTMS on previous testing days might interfere with learning during the subsequent 3 days of training on the feature task. First we considered thresholds for the feature task before and after training for 3 groups of participants: (i) those given rTMS to PPC (n=12), (ii) those given rTMS to LO (n=12) and (iii) participants (n=7) who did not receive TMS (including data from four participants of Exp. 2 of Chang *et al* [S3]). There were clear improvements in task performance after training ($F_{1,28}$=103.2, p<.001), but no differences between the different groups ($F_{2,28}$<1, p=.48) nor an interaction ($F_{1,2}$=1.1, p=.37). In addition, we fit the learning functions for each individual and compared the rate parameter (k) between groups, finding no evidence for differences between groups ($F_{2,28}$<1, p=.43). (NB all the data presented here represent behavioural performance where at least 24 hours had elapsed since rTMS.)

Fig. S3: Horizontal eye vergence response functions (Main Fig. 4)

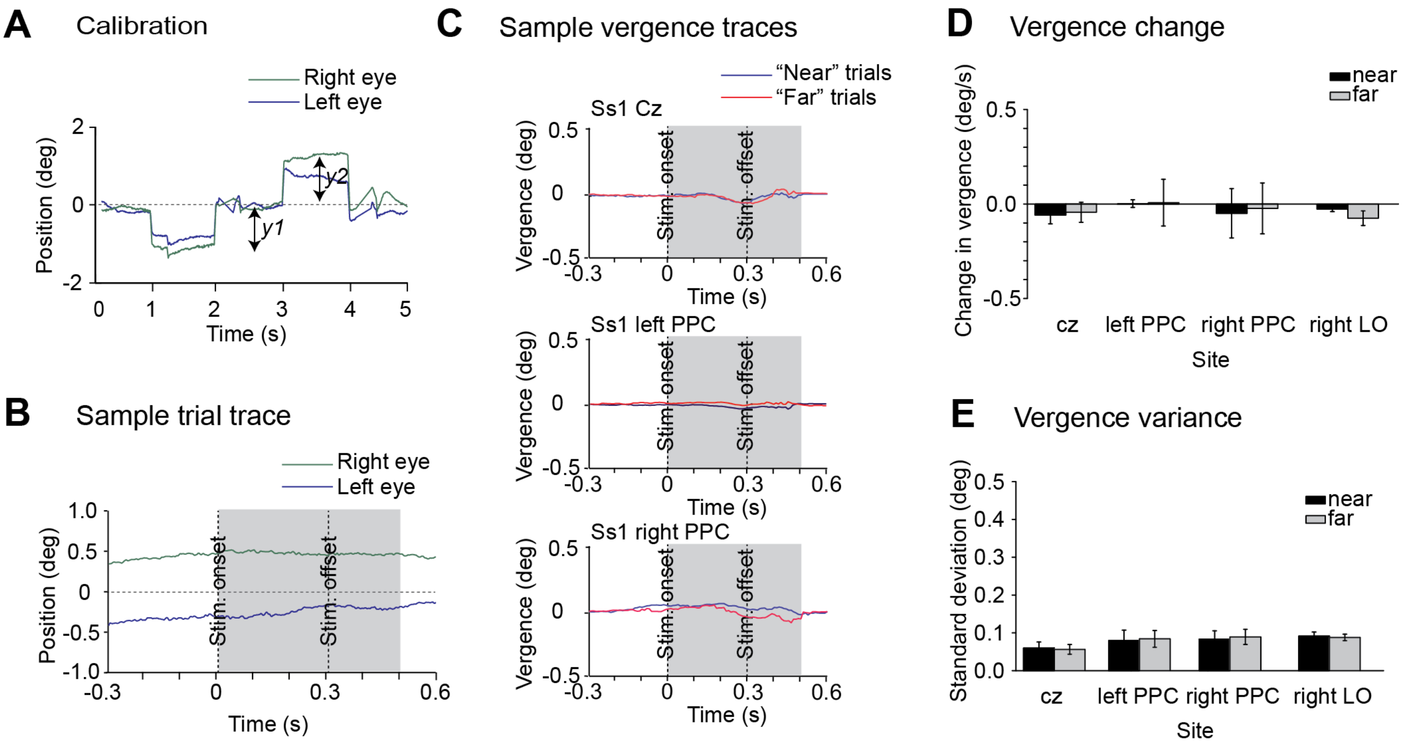

Eye-movement data presented for Cz, left- and right- PPC were measured pre-training, and eye-movement data presented for right LO were measure post-training, corresponding to the critical periods during which we observed task performance changes with TMS.

(A) Horizontal eye position data from a sample calibration window from one observer. Calibration data from each run were used to correct drift from centre (as indicated by the dashed line), and to compute a gain parameter corresponding to (y1 + y2) / 2. Data from each window of trials that followed each calibration block were recalibrated using these parameters.

(B) Horizontal eye position data from a sample rTMS trial from one observer. The portion of trial during which rTMS pulses were delivered is shown by the gray box. Stimulus onset and offset are indicated by the vertical dashed lines.

(C) Mean event-related horizontal vergence trace from a sample run from one observer. The interval of 300 ms prior to stimulus onset to 300 ms after stimulus offset is shown. Horizontal vergence angle was computed as the right horizontal eye minus the left horizontal eye. Thus, negative values for vergence correspond to positions that are nearer than the screen. The data are shown separately for near *vs*. far trials across stimulation sites. The portion of this window during which rTMS pulses were delivered is shown by the gray box. Stimulus onset and offset are indicated by the vertical dashed lines.

(D) To quantify any change of vergence that might take place during a trial, we fit a line to the eye vergence data of individual trials during the 300 ms window corresponding to stimulus presentation. We thereby quantified vergence changes on each trial in terms of the gradient (β) of the best fitting (least-squares) linear model to the data. Thereafter we compared the gradient terms for TMS vs. no TMS, finding no differences. In addition, during trials with rTMS stimulation, vergence did not vary depending on trial type (near/far), $F_{1,3}<1$, $p>.5$, or, stimulation site, $F_{3,6}<1$, $p>.5$. These data suggest that changes in vergence do not provide an account for the rTMS effects observed in our experiments.

(**E**) As a complementary analysis to **D**, we also computed the variance (standard deviation) in vergence position during the 300 ms window corresponding to stimulus presentation for each trial. We observed no differences in vergence variability between trials during which rTMS stimulations were applied and trials during which no stimulation was applied. Additionally, there were no differential effects of trial type (near/far), $F_{1,3}<1$, $p=.45$, or stimulation site, $F_{3,6}=1.2$, $p=.38$ on vergence variability.

Fig. S4: Response times (Main Fig. 4)

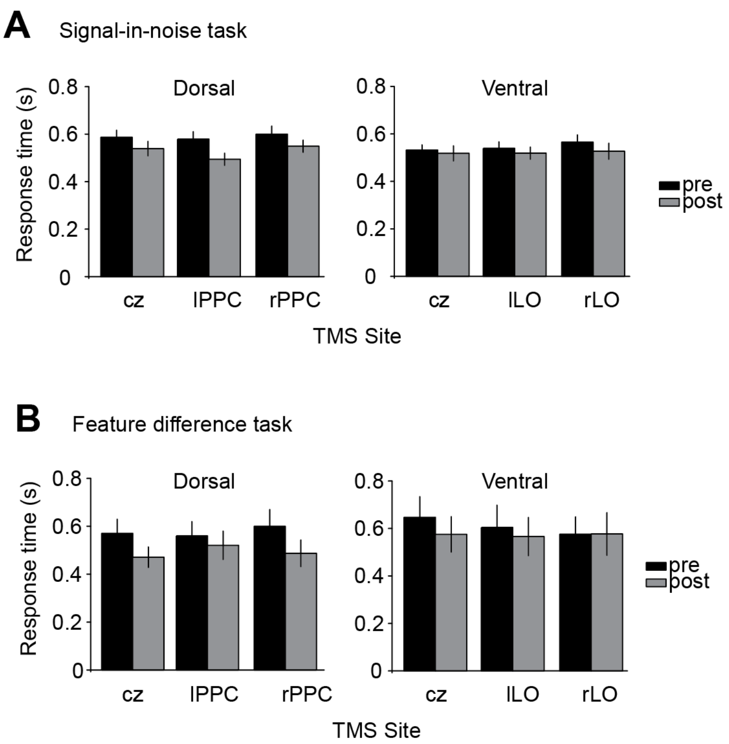

We analysed response time data from participants stimulated over both dorsal and ventral cortex, before and after training for both the (**a**) signal-in-noise and (**b**) feature difference tasks. Response times for the signal-in-noise task improved after training, $F_{1,22}$=6.02, p=.023, but did not differ between region of interest (PPC, dorsal *vs*. LO, ventral) ($F_{1,22}$<1, p=.48), or site (Cz, left, right) ($F_{2,44}$=2.71, p=.078), nor were there any significant interactions. Response times for the feature difference task improved after training ($F_{1,7}$=7.29, p=.031), but did not differ between region of interest (PPC, dorsal *vs*. LO, ventral), ($F_{1,7}$<1, p=.99) or site (Cz, left, right) ($F_{2,14}$<1, p=.89), nor were any interactions significant. These data suggest that there is little systematic effect of TMS over the sites of interest for response times on this task. This is expected as participants were instructed to perform the near threshold task accurately, and speed of response was not emphasised. Response times differ from the effects on threshold, thus do not appear to provide any form of explanation for the main experimental data reported in the paper.

Table S1: Mean and SEM of the Talaraich coordinates for the stimulation sites of interest:

		Left hemisphere			Right hemisphere		
**Region**	**N**	**x**	**y**	**z**	**x**	**y**	**z**
PPC	9	–34.1 (2.3)	–62.3 (4.5)	42.3 (2.3)	29 (1.6)	–66.5 (3.2)	43.3 (1.7)
hMT+	6	–44.6 (1.7)	–64.4 (1.1)	6.6 (2.2)	42.7 (1.2)	–57.7 (1.3)	2.0 (1.3)
LO	18	–40.9 (0.6)	–68.3 (1.0)	–4.1 (1.0)	41.5 (0.5)	–65.0 (1.0)	–3.7 (1.0)

Supplemental Experimental Procedures

Participants

Participants (n=62) age ranged from 18 to 32 years (mean=22). All had normal or corrected-to-normal vision, were screened for stereo deficits, epilepsy or other neurological disorders in themselves or in their family, and provided written informed consent in line with local ethical review and approval of the work.

Stimuli

Stimuli were random dot stereograms (RDS) (**Fig. 1**) surrounded by a grid of background squares (size = 0.5 deg), designed to provide a background reference and promote stable vergence. The RDS depicted a central target (diameter = 6 deg) surrounded by an annulus (“the surround”, diameter = 12 deg). Individual dots subtended 0.15 deg and there were 6 dots/deg^2. Participants judged the position (in front / behind) of the central target relative to the surround. Task difficulty varied in one of two ways: 1) Signal-in-noise task: the target plane had disparity ± 6 arcmin (crossed or uncrossed) and we varied the percentage of dots defining the target (signal) relative to noise dots that had a random disparity within ± 12 arcmin. 2) Feature difference task: the surround had a disparity of 12 arcmin (crossed or uncrossed) and we varied the disparity between the target and surround in fine steps. For initial parietal stimulations (n=12) stimuli were presented on a 22 inch ViewSonic VX2260WM LCD display viewed through red/green anaglyphs. All subsequent experiments (including replications of parietal effects: **Fig. S2a,b**) employed a haploscope in which the two eyes viewed separate 22 inch Samsung (2233) LCD displays through front-silvered mirrors. Viewing distance was 50cm. Graphics rendering and anti-aliasing was implemented by an nVidia Quadro 4000 graphics card to display 1280 x 1024 pixels at 60 Hz on each display. Stimulus duration was 0.3s.

rTMS

Stimulations were applied using a 70 mm figure-of-eight coil connected to a MagStim Rapid2 stimulator (MagStim, Whitland, UK) over left and right PPC, left and right LO, and Cz. The position of the coil was identified based on the 10-20 EEG coordinate system (left PPC (P3), right PPC (P4)), shown previously to correspond to posterior IPS (see **Table S1**), or using the Brainsight (Rogue Research) TMS-MRI coregistration system (left and right LO, hMT+/V5). The lateral occipital complex (LOC), the human motion complex (hMT+/V5) and retinotopic visual areas were defined using standard procedures [S4]. The 10-20 EEG coordinate system was also used to localise Cz. For nine participants, we obtained high-resolution anatomical (1 mm) scans with cod liver oil capsules (500 mg) positioned at electrode positions P3 (left PPC) and P4 (right PPC) of the 10-20 EEG coordinate system (**Fig. 2**; **Table S1**).

For all stimulation sites, the coil was placed tangential to the head with the handle pointing posteriorly (for parietal and Cz stimulation) or superiorly (for LO stimulation and hMT+/V5). Online stimulation was given at 10 Hz (5 pulses synchronised with stimulus onset) with a fixed intensity of 60% of the stimulator’s maximum output [S5] for all sites of interest. Comparing task performance under TMS across areas could be problematic if the efficacy of the TMS perturbation varies between areas (e.g. due to differences in the distance of the area from the skull and/or differences in skull/muscle thickness). However, in our case, we assessed TMS effects of two different tasks within the same area, as well as before vs. after training within the same area. We applied TMS at a level that is compatible with other published work in this field, and found that the same amount of TMS produced dissociable effects on our two tasks. This fixed stimulator intensity resulted in performance reductions in both PPC and LO, meaning that this protocol was sufficiently sensitive to detect neural effects in different areas before and after training. That is, the same amount of TMS had an effect in PPC before training, but no effect after training; in contrast to TMS to ventral cortex that was effective after-, but not before-, training. Further performance disruption was comparable ($t_{22}<1$, $p=.497$) for PPC TMS before training (18.76% ± 5.73 SEM) and LO TMS after training (24.92% ± 6.84 SEM). No participants reported phosphenes over any stimulation site. To prevent overheating, the TMS coil was replaced after each run. The TMS protocol may induce a mixture of effects time-locked to stimulus presentation and carry-over effects.

The rTMS experiment consisted of three phases: 1) Pre-training tests carried over three separate days, each testing a different stimulation site (e.g., left PPC, right PPC, Cz or lLO, rLO, Cz or rhMT+, lhMT +, Cz). Within each session, the participant was tested on two runs (208 trials in total) of each of the signal-in-noise and feature difference tasks. rTMS was only applied during one task (i.e., signal-in-noise or feature difference task), and this was always the second task performed to avoid TMS carryover effects. 2) Training on a task that comprised 21 runs (2184 trials) completed over three consecutive days. 3) Post-training tests carried over three separate days which were identical to those completed pre-training. The order of stimulation sites was counterbalanced across participants, but was fixed between pre- and post-training tests for each observer. For each test and training run, task difficulty was adjusted according to two interleaved staircases determining thresholds at the 82%-correct level. For each participant, the threshold for a given run was computed as the mean of the thresholds from each staircase.

fMRI

Region of interest localizer imaging data for the participants were acquired at the Birmingham University Imaging Centre using a 3-tesla Philips MRI scanner with an eight-channel head coil. Blood oxygen level–

dependent signals were measured with an echo-planar sequence (TE 35 ms; TR 2000 ms; 2.5 × 2.5 × 3 mm, 32 slices). For each participant, we additionally acquired a high-resolution (1 mm) anatomical scan. fMRI data were analysed with BrainVoyager QX (BrainInnovation B.V.). For each participant, we transformed anatomical data into Talairach space. Functional data were preprocessed using three-dimensional motion correction, slice time correction, linear trend removal and high-pass filtering (three cycles per run cut-off). LO was defined as the set of contiguous voxels in the lateral occipitotemporal cortex that showed significantly stronger activation for intact than scrambled images, consistent with previous reports [S6].

Eye recording and analysis

Binocular eye movements were recorded using an Eyelink 1000 remote video tracker (SR Research), with sampling rate 500 Hz. The system has a stated accuracy of 0.25 deg and resolution of 0.01 deg RMS. The tracker viewed participants' eyes through the (infrared transmitting) cold mirrors of the stereoscope.

On each run, observers were instructed to maintain fixation on a square marker (0.5 deg on each side) with horizontal and vertical nonius lines (0.3 deg in length). This square marker was centred and present throughout the entire run, but shifted horizontally (e.g., centre, +1 deg horizontal, centre, –1 deg horizontal, centre) during each calibration block that occurred at the start of each run, and once every 10 trials thereafter (11 blocks per run). Each calibration block lasted 5 seconds during which the configuration of fixation shifts was selected randomly between [0 –1 0 1 0] or [0 1 0 –1 0], where zero indicates a centred fixation marker, and ±1 indicate 1 deg shifts to the horizontal right or left, respectively.

To analyse the eye movement data of a run, we first converted raw gaze positions to degrees of visual angle. The time series data were then preprocessed by removing any data that corresponded to periods of blinks (average 7% of a given run for both trials with and without TMS) or saccades (average 12% of a given run for both trials with and without TMS), as identified by the EyeLink inbuilt detection functions. We followed this with a manual inspection of the data to ensure there were no additional blinks or saccades that were not detected by these functions. Any periods during which tracking was lost in one or both eyes or during which data were excessively noisy (due to instability of the eyetracker in determining pupil-corneal reflections) were additionally discarded (<1% of a given run). All removal of data were performed "blind" to experimental conditions, and required the agreement of two of the authors.

The remaining data were then subject to drift and gain correction using data from the calibration blocks. For each calibration block, we computed average centre coordinates of the observer's gaze from periods in which the fixation marker was centred, and a gain parameter that corresponded to the average horizontal amplitude of the observer's gaze during shifts of the fixation marker, (y1 + y2)/2 (**Fig. S3a**). The parameters from each calibration block were then used to recalibrate positional data from the window of trials that preceded it. Next, the recalibrated data were segregated into trial windows that included the period from 300 ms prior to stimulus onset to 300 ms after stimulus offset. Horizontal vergence angle was computed as the right horizontal eye position minus the left horizontal eye position (in relation to fixating at the centre of the screen). Thus, negative vergence values correspond to positions that are nearer relative to fixation)**.** Vergence changes and variability were computed across the 300 ms trial window corresponding to stimulus presentation only (**Fig. S3 d, e**).

Statistical Analyses

Statistical analysis was conducted in SPSS (SPSS Inc, Chicago, Ill). We analysed raw threshold values using repeated-measures ANOVAs, and applied Greenhouse-Geiser correction where appropriate. We used bonferonni corrected *t*-tests for post-hoc analyses.

Supplemental References

S1. Silvanto, J., Lavie, N., & Walsh, V. (2005). Double dissociation of V1 and V5/MT activity in visual awareness. *Cerebral Cortex,* 15(11), 1736–1741.

S2. Walsh, V., Ashbridge, E., & Cowey, A. (1998). Cortical plasticity in perceptual learning demonstrated by transcranial magnetic stimulation. *Neuropsychologia*, 36(4), 363–367.

S3. Chang, D. H. F., Kourtzi, Z., & Welchman, A. E. (2013). Mechanisms for extracting a signal from noise as revealed through the specificity and generality of task training. *Journal of Neuroscience*, 33(27), 10962–10971

S4. Preston, T. J., Kourtzi, Z., & Welchman, A. E. (2009). Adaptive estimation of three-dimensional structure in the human brain. *Journal of Neuroscience*, 29(6), 1688–1698.

S5. Mevorach, C., Humphreys, G. W., & Shalev, L. (2006). Opposite biases in salience-based selection for the left and right posterior parietal cortex. *Nature Neuroscience*, 9(6), 740–742.

S6. Kourtzi, Z., & Kanwisher, N. (2001). Representation of perceived object shape by the human lateral occipital complex. *Science*, 293(5534), 1506–1509.
